# Characterization of a novel monoclonal antibody candidate that targets bacterial GAPDH and protects neonatal mice from infections caused by *Streptococcus pneumoniae* or *Streptococcus agalactiae*

**DOI:** 10.1128/aac.00666-25

**Published:** 2026-01-14

**Authors:** Carolina Fernandes, Filipa Lemos, Liliana Curado, Bruno L. Victor, Maelenn Chevreuil, Inês Machado, Sofia Teixeira, Maria do Carmo Koch, Marta Vieira, Patrick England, Pedro Madureira, Pedro Castanheira

**Affiliations:** 1 Immunethep, Biocant Park 840245 , Cantanhede, Portugal; 2 ICBAS – Instituto de Ciencias Biomédicas Abel Salazar, Universidade do Porto 26706, Porto, Portugal; 3 Accelbio: Collaborative Laboratory to Foster Translation and Drug Discovery 637393 , Cantanhede, Portugal; 4 Plateforme de Biophysique Moléculaire, Institut Pasteur, CNRS UMR3528 27058, Paris, France; 5 Serviço de Imuno-hemoterapia, Centro Hospitalar e Universitário de São João 285211, Porto, Portugal; The Peter Doherty Institute for Infection and Immunity, Melbourne, Victoria, Australia

**Keywords:** GAPDH, *Streptococcus*, monoclonal antibody, bacterial sepsis

## Abstract

Glyceraldehyde-3-phosphate dehydrogenase (GAPDH) is a highly conserved bacterial enzyme essential for glycolysis, yet it also plays important non-metabolic, “moonlighting” roles, including host immunomodulation. Here, we describe the generation and characterization of a monoclonal antibody (mAb01) that targets extracellular GAPDH from *Streptococcus agalactiae* (GBS) and *Streptococcus pneumoniae*. Using bio-layer interferometry (BLI) and surface plasmon resonance (SPR), we demonstrate that mAb01 binds with high affinity, exhibiting dissociation constants in the low nanomolar range for both antigens. Functionally, mAb01 significantly improves survival in a neonatal murine model of sepsis caused by either GBS or *S. pneumoniae*, two relevant neonatal pathogens. The addition of mAb01 to *ex vivo* cultures of human peripheral blood infected with GBS or *S. pneumoniae* induced a significant decrease in bacterial replication, further supporting its protective potential. These results provide the first demonstration of a high-affinity anti-GAPDH monoclonal antibody that is effective in both *in vivo* and *ex vivo* models of streptococcal infection.

## INTRODUCTION

*Streptococcus* species are among the leading causes of both healthcare- and community-acquired bacterial infections worldwide. These pathogens are associated with a broad spectrum of diseases, including pneumonia, neonatal sepsis, meningitis, endocarditis, pharyngitis and tonsillitis, otitis and bacteremia (blood stream infection), collectively responsible for millions of deaths worldwide (1–3).

The widespread use of antibiotics has contributed to the emergence and global dissemination of antimicrobial resistance (AMR), and Streptococcus species are no exception. The major human pathogenic streptococci*—S. pneumoniae*, *S. pyogenes* (GAS: Group A *Streptococcus*), and *S. agalactiae* (GBS: Group B *Streptococcus*)—exhibit alarming levels of resistance not only to penicillin but also to key alternative treatments, including macrolides, quinolones, and lincosamides (3). AMR has become a significant factor contributing to treatment failure.

Vaccination is a proven strategy for combating AMR, yet current vaccines targeting *Streptococcus* are limited to *S. pneumoniae* relying on capsular polysaccharides. However, these vaccines provide incomplete serotype coverage, protecting against only 20 out of more than 90 serotypes. This limitation has led to an increased incidence of serotypes not covered by the available vaccines (4, 5). Additionally, reports of invasive infection caused by non-encapsulated *S. pneumoniae* have risen in recent years, likely due to selective pressure exerted by existing vaccines (6–8).

To address these challenges, we have been investigating an alternative approach targeting the glycolytic bacterial enzyme glyceraldehyde-3-phosphate dehydrogenase (GAPDH). Immunomodulatory GAPDH was shown to be released by GBS and *S. aureus* and triggers an early production of interleukin-10 (IL-10) (9–11). IL-10 suppresses key inflammatory responses, impairing the host’s ability to control bacterial replication effectively (9, 12, 13). Signaling through Toll-like receptor 2 (TLR2) is essential for the early IL-10 production observed in GBS infections (9, 10). Immunization with recombinant bacterial GAPDH (bGAPDH) has been shown to confer significant protection in mice models of infection caused by GBS (13, 14), *S. aureus* (11), and *S. pneumoniae* (10). These findings suggest that GAPDH-mediated immune evasion is a conserved virulence mechanism across multiple bacterial species.

However, the high sequence conservation between bacterial and human GAPDH poses a challenge for a human vaccine development. To overcome this, we developed an experimental immunogenic formulation (IMTP_vac_1804), which consists of synthetic peptides derived from bGAPDH regions with low sequence similarity to human GAPDH and high solvent exposure on the bacterial protein surface. Maternal immunization with such formulation resulted in significant protection of offspring against GBS and *S. aureus* (11). Moreover, passive immunization with anti-bGAPDH or with IgG raised against IMTP_vac_1804 conferred protection against lethal infections with GBS or *S. aureus* (11, 13). In both active and passive immunization models, protection correlated with reduced IL-10 levels and decreased bacterial loads in infected organs (11, 13), supporting the role of GAPDH in bacterial virulence.

Preventive vaccines have proven to be invaluable in reducing the incidence of infectious diseases, but new therapeutic agents that can be used in acute settings as an alternative to antibiotics are also urgently needed. Therefore, we describe for the first time the characterization of a monoclonal antibody targeting *Streptococcus* GAPDH, demonstrating nanomolar affinity for GAPDH from both GBS and *S. pneumoniae*. Importantly, this monoclonal antibody enhances the survival of mice challenged with lethal inocula of either pathogen. These findings underscore the pivotal role of GAPDH in bacterial immune evasion and pathogenesis, reinforcing the necessity of neutralizing this enzyme to improve infection control. Given the urgent need for alternative strategies to combat antibiotic-resistant infections, the development of monoclonal antibodies targeting bGAPDH represents a promising immunotherapeutic approach that could complement existing treatments and enhance host defense mechanisms.

## MATERIALS AND METHODS

### Monoclonal antibody production and purification

Hybridoma cells were cultured in RPMI-1640 medium with 10% FBS and 1% penicillin-streptomycin at 37°C and 5% CO₂. After expansion, supernatants containing secreted antibodies were clarified by centrifugation (500 × *g*, 10 min).

Antibodies were purified using Protein G affinity chromatography (Cytiva). Clarified supernatants were loaded onto columns equilibrated with 20 mM sodium phosphate buffer (pH 7.0) at 1 mL/min. After washing, antibodies were eluted with 0.1 M glycine-HCl (pH 2.7) and immediately neutralized with 1 M Tris-HCl (pH 9.0). Eluates were buffer-exchanged into PBS using Amicon Ultra-15 centrifugal filters (30 kDa cutoff, Merck).

Protein concentration was measured at 280 nm (NanoDrop, Thermo), and purity was assessed by SDS-PAGE under reducing and non-reducing conditions.

### Production and purification of recombinant GAPDH

Recombinant GAPDH (rGAPDH) from *Streptococcus pneumoniae* (Q8CWN6), *Streptococcus agalactiae* (Q8DXS8), and human (P04406) was expressed in *E. coli* BL21 Star (DE3) using codon-optimized genes cloned into pET28a vectors. Constructs encoded either an N-terminal 6×His tag with an HRV 3C protease site (6His_3C_rGAPDH) or a non-cleavable C-terminal 6×His tag (rGAPDH_6His). Plasmids were sequence-verified before transformation.

Transformants were selected on LB agar with kanamycin (50 µg/mL), grown overnight, diluted 1:50 in fresh LB/Kan, and cultured at 37 °C to OD_600_ ≈ 0.5. The temperature was lowered to 18°C, and 0.1 mM IPTG was added. Expression proceeded overnight. Cells were harvested (6,000 × *g*, 20 min), resuspended in phosphate buffer (20 mM, pH 7.4, 500 mM NaCl, 15 mM imidazole) with lysozyme, and then frozen at −20°C.

Lysates were treated with MgCl_2_ and DNase I, clarified by ultracentrifugation (50,000 × *g*), filtered (0.22 µm), and purified by IMAC using a HisTrap HP column. rGAPDH eluted at 150 mM imidazole. For tag removal, 6His_3C_rGAPDH was buffer exchanged, digested with HRV 3C protease (1:100 molar ratio), and repurified by IMAC; cleaved protein (rGAPDHΔHis) was collected from the flowthrough.

All proteins were purified by SEC (Superdex 200 26/600 pg) in PBS, quantified by A_280_, flash-frozen, and stored at −80°C. Prior to use, a polishing SEC step was performed on a Superdex 200 Increase 10/300 GL column.

### Protein quality control

Protein quality control for both mAb01 and rGAPDH was conducted following the guidelines recommended by the P4EU and ARBRE-MOBIEU European networks (15).

#### Sample homogeneity and quantification

Dynamic light scattering (DLS) analysis was performed using a DynaPro Plate Reader III (Wyatt, Santa Barbara, CA, USA) to assess sample homogeneity and detect potential aggregates. A 20 µL sample was loaded into a 384-well microplate (Corning, ref. 3540, New York, USA), with 10 acquisitions of 5 s each at 20°C, monitored using DYNAMICS software (version V7.10.0.21, Wyatt, Santa Barbara, CA, USA). Each measurement was repeated three times. DLS analysis was also performed after centrifugation at 27,000 × *g* for 15 min at 4°C.

#### Detection of DNA contamination and protein quantification

Protein quantification at 280 nm was performed by recording a full absorbance spectrum from 240 to 340 nm. Measurements were conducted at 20°C using a 1 cm quartz cuvette (Hellma, ref. 105.202-QS.10, France) in a JASCO V-750 spectrophotometer (JASCO Corporation, Japan). DNA contamination was assessed at 260 nm, and light scattering was recorded at 340 nm. Baseline subtraction at 340 nm was applied using Spekwin32 software (F. Menges, Version 1.72.2, 2016, http://www.effemm2.de/spekwin/) to ensure accurate protein concentration calculations. Cuvettes were cleaned with 2% Hellmanex, followed by rinsing with water and 70% ethanol.

#### Protein integrity and purity

Protein integrity and purity were analyzed using a Bruker UltrafleXtreme MALDI-TOF/TOF mass spectrometer. A 15 µL protein sample was purified using a ZipTip C4 and eluted onto an MTP 384 ground steel target plate (Bruker-Daltonics, Germany) with 2 µL of a matrix solution containing 20 mg/mL α-cyano-4-hydroxycinnamic acid (HCCA) in 50% acetonitrile (ACN) and 0.1% trifluoroacetic acid (TFA). Spectra were acquired using FlexControl software (Bruker-Daltonics, Germany) in positive ion linear mode. External calibration was performed in the *m*/*z* range of 30–210 kDa using the Protein II standard (Bruker-Daltonics, Germany), and data analysis was conducted with FlexAnalysis software (Bruker-Daltonics, Germany).

#### Oligomerization state analysis

The oligomerization state of the proteins was determined by size-exclusion chromatography (SEC) coupled with triple detection, including a UV detector, refractometer, static light scattering (7° and 90°), and a viscometer, using an OMNISEC RESOLVE and REVEAL system (Malvern Panalytical, UK). The SEC column and detectors were equilibrated with PBS filtered through 0.2 µm filters (Filtermax TPP). Samples were injected into a Superdex 200 Increase 10/300 GL column (Cytiva) at 20°C and eluted at a flow rate of 0.4 mL/min. A 100 µL sample at 3.4 mg/mL was analyzed, and external calibration was performed using bovine serum albumin (BSA) (Sigma, ref. A1900) injected at 10 µL with a concentration of 18.3 mg/mL. The refractive index, static light scattering, and viscosity measurements were processed using OMNISEC software (version V11.32, Malvern Panalytical, UK) to determine the mass-average molecular mass and intrinsic viscosity.

### Enzyme-linked immunosorbent assay

ELISAs were performed in 96-well MaxiSorp plates (NUNC) coated overnight at 4°C with 5 µg/mL of 6His-tagged bacterial or human rGAPDH in PBS. Plates were washed (PBS + 0.05% Tween-20) and blocked with 1% BSA in PBS for 2.5 h at room temperature, followed by a second wash.

A threefold serial dilution of mAb01 (eight points, starting at 3 µg/mL in blocking buffer) was added in duplicate alongside blanks and incubated for 1 h at room temperature. Plates were washed and incubated with 50 µL of goat anti-mouse IgG–HRP (1:5,000, Southern Biotech) for 45–60 min. After washing, 50 µL of TMB Ultra substrate (Thermo) was added and incubated in the dark (~5 min). Reactions were stopped with 50 µL of 1 M H₂SO₄, and absorbance at 450 nm was measured (Thermo Multiskan FC). Blank-corrected values were fitted using a four-parameter logistic curve (GraphPad Prism v9) to determine EC₅₀.

For quantifying antibody in hybridoma supernatants, plates were coated with 2 µg/mL anti-mouse IgG in PBS. A standard curve (mouse IgG1κ-UNLB, 10 nM starting concentration, threefold dilution) and fivefold-diluted supernatants were added. Detection was performed using goat anti-mouse κ-HRP (Southern Biotech) as above.

### Western blotting

Proteins were mixed with Laemmli buffer (Bio-Rad) containing 2.5% 2-mercaptoethanol and denatured at 90°C for 10 min. Samples were resolved on 15% acrylamide Tris-glycine SDS gels and transferred to PVDF membranes (Cytiva) in transfer buffer (50 mM Tris, 40 mM glycine, 1.73 mM SDS, 20% methanol) at 400 mA for 1.5 h under refrigeration.

Membranes were blocked with 5% FBS in TBS-T (137 mM NaCl, 20 mM Tris, 0.1% Tween-20) and incubated with primary antibodies diluted in TBS-T for 1 h at room temperature. After washing with TBS-T, membranes were incubated with HRP-conjugated secondary antibodies for 1 h, followed by further washes.

Signals were developed using chemiluminescent ECL substrate (Bio-Rad), and bands were imaged using a digital imaging system (VWR).

### Biolayer interferometry

mAb01 binding to rGAPDH was monitored by biolayer interferometry (BLI) using an Octet Red384 instrument (Sartorius). Anti-mouse Fc (AMC) biosensors or Ni-NTA biosensors were loaded with mAb01 and GBS rGAPDH-6His, respectively.

For the experiments with captured mAb01 (using AMC biosensors), after a 2-min baseline step (incubation in PBS + 1% BSA), the biosensors were loaded with mAb01 at 2 μg/mL for 1 min, followed by another baseline step. Subsequently, the biosensors were incubated in wells containing serial twofold dilutions of the respective rGAPDH proteins (7.8–500 nM), and the BLI association signals were recorded in real-time for 30 min. Finally, the biosensors were incubated in wells containing buffer to monitor the dissociation of the complexes formed for 1 h, before being regenerated for further use in replicate experiments. The regeneration protocol, comprising three consecutive 30-s washes in 10 mM Gly-HCl (pH 1.7), could be applied up to eight times over 2 days without losing loading capacity.

For the experiments with captured GBS rGAPDH-6His (using NTA biosensors), after a 2-min baseline step (incubation in PBS + 1% BSA), the biosensors were activated by a 3-min incubation in 10 mM NiCl₂ (Sigma-Aldrich), followed by a second baseline step before being loaded for 10 min with GBS rGAPDH-6His at 2 μg/mL, followed by another baseline step. Subsequently, the biosensors were incubated in wells containing serial two-fold dilutions of mAb01 (0.625–40 nM), and the BLI association signals were recorded in real-time for 1 h. Finally, the biosensors were incubated in wells containing buffer to monitor the dissociation of the complexes formed for 1 h, before being regenerated for further use in replicate experiments. The regeneration protocol, comprising three consecutive 30-s washes in 10 mM Gly-HCl (pH 1.7), followed by three additional 30-s washes in 0.5 M EDTA, could be applied up to eight times over 2 days without losing loading capacity.

Specific rGAPDH binding curves were obtained by subtracting the non-specific signals measured on unloaded biosensors used as control references. All experiments were performed at 25°C in PBS supplemented with 1 mg/mL BSA to minimize nonspecific binding, using 96-well plates (Greiner Bio-One) filled with 300 μL per well, and a shaking speed of 1,000 rpm. Data were processed using Octet Data Analysis High Throughput (HT) software, Release 11 (Sartorius).

### Surface plasmon resonance analysis

SPR experiments were performed on a Biacore T200 instrument (Cytiva) equilibrated at 25°C in PBS supplemented with 1 mg/mL BSA. The interaction between mAb01 and rGAPDH was monitored in both orientations, using either a Sensor Chip CM5 (Cytiva), where mAb01 was captured via immobilized anti-mouse Fc IgG (Cytiva), or a Sensor Chip NTA (Cytiva), where GBS rGAPDH_6His was captured via its hexahistidine tag.

mAb01 (100 μg/mL) was captured by anti-mouse IgG immobilized on a Sensor Chip CM5 for 180 s at 5 μL/min, reaching a surface density of 400–450 RU (resonance units; 1 RU ≈ 1 pg/mm²). The different rGAPDH proteins (20 and 200 nM) were then injected at 20 μL/min, with association monitored for 17.5 min, followed by buffer injection for 20 min at 20 μL/min to track dissociation. Regeneration was achieved by injecting 10 mM Gly-HCl pH 1.5 for 1 min at 5 μL/min.

For full kinetic analysis, an NTA sensor chip was used. After a 5 min loading step with NiCl₂ at 5 μL/min, GBS rGAPDH_6His was captured at three different densities (80, 125, and 1,250 RU) in flow cells (Fc) 2, 3, and 4, respectively, while flow cell 1 served as a reference. GBS rGAPDH_6His was loaded at 2 μg/mL for 150 s in Fc 2, and at 10 μg/mL for 80 s and 150 s in Fc 3 and Fc 4, respectively. mAb01 was then injected at 20 μL/min for 17.5 min across all flow cells to monitor association, followed by buffer injection at 20 μL/min for 20 min to track dissociation. Regeneration was achieved by injecting EDTA 0.5 M for 1 min at 5 µL/min.

Data were processed using Biacore T200 Evaluation Software v3.1 and Biacore BiaEvaluation v3.2.

### Determination of GAPDH enzymatic activity

The enzymatic activity of GBS or *S. pneumoniae* rGAPDH was measured by using a commercial kit from Abcam (cat. #ab204732) according to the manufacturer’s instructions.

The activity of GBS and *S. pneumoniae* GAPDH was assessed using 1 μg and 2 μg of recombinant protein per reaction, respectively. To evaluate the effect of mAb01 or the respective isotype control on the GAPDH activity, antibodies were added at a 16-fold excess relative to the amount of recombinant GAPDH tetramer. The final volume of the reaction was 50 μL. The absorbance was measured every 30 s for 20 min.

Hydrogen peroxide was used as a positive control for complete inhibition of GAPDH enzymatic activity. For this control, the protein used in this assay was incubated with 1 mM of hydrogen peroxide for 15 min at room temperature, prior to the determination of the activity.

### Bacterial strains and growth conditions

*S. pneumoniae* and GBS clinical isolates were kindly provided by the Microbiology Department of Centro Hospitalar Universitário de Santo António (Porto, Portugal). For infection challenge experiments, bacteria were grown to log phase. GBS was cultured in Todd-Hewitt (TH) broth (Oxoid) at 37°C with shaking, while *S. pneumoniae* was cultured in TH broth supplemented with 2% yeast extract and 10 mg/L choline chloride, without shaking. Multi-Locus Sequence Typing (MLST) identified the *S. pneumoniae* isolate as ST63, serotype 15A, while the GBS isolate was ST23, serotype III.

### Animals

Male and female C57BL/6J mice (6–8 weeks old) were purchased from Charles River Laboratories. The experiments were performed at the animal facilities of the Institute for Research and Innovation in Health (i3S) and the Center for Neuroscience and Cell Biology of Coimbra (CNC). Challenging infections were conducted at the Animal Biosafety Level 2 (ABSL2) facility under the conditions specified by Portuguese Directive 102-A/2020, which included negative pressure, 40 air changes per hour, a temperature range of 20–24°C, a 12-h light/dark cycle, and 55% ± 10% relative humidity. Animals were housed in individually ventilated polycarbonate (type IIL) cages under negative pressure. Food (Mucedola Diet) and distilled water were autoclaved and provided *ad libitum*.

Procedures were designed to minimize animal suffering in accordance with institutional standard operating procedures. Throughout the experiment, newborn mice remained with their mothers.

### Challenging infections

Neonatal mice (24 h old) were passively immunized with 200 μg of mAb01 or control mAb (BioXcel) subcutaneously (s.c.) and challenged with either 1 × 10^6^ CFU of *S. pneumoniae* or 3 × 10^6^ CFU of GBS (administered s.c. in a maximum volume of 40 μL) 24 h after the mAb treatment. Survival was monitored daily for 12 days post-infection. Throughout the experiment, mouse pups remained with their mothers.

### *Ex vivo* model of bacteremia

Human peripheral blood was obtained from the Immuno-Hemotherapy Department of São João Hospital and University Center (CHUSJ) under informed consent.

Freshly collected peripheral blood was diluted twofold with the same volume of RPMI medium (Gibco) supplemented with 1% (vol/vol) 1 M HEPES pH 7.2 (Sigma-Aldrich). Cells were incubated at 37°C with 5% CO₂ for specific timepoints with a multiplicity of infection (MOI) of 10 for both GBS and *S. pneumoniae*. Medium was supplemented with 10 µg of either monoclonal or control IgG (InvivoMab, isotype control with unknown specificity). After incubation, serial dilutions of the cell culture were prepared in sterile PBS for CFU analysis. For GBS, dilutions were plated on TH agar, while for *S. pneumoniae*, dilutions were plated on blood agar.

### Statistical analysis

Statistical analysis was performed using GraphPad Prism version 10.4.1. Survival curve significance was evaluated using the log-rank test. Outliers were identified as values exceeding mean ± 2 × SD. A parametric *t*-test with Welch’s correction was used to assess differences between treatments. *P*-values < 0.05 were considered statistically significant.

### ELISA detection of surface GAPDH

For both *S. pneumoniae* and GBS, 1 × 10^7^ CFU were first incubated for 1 h at 4°C with swine IgG (Swine IgG-UNLB, cat# 0137-01; CliniSciences) to block any Fc-binding proteins at the bacterial surface. Then, each bacterium was incubated overnight at 4°C with either mAb01 or mouse IgG1 isotype control (cat# BE0083; InVivoMAb) at 12.5 µg/mL, and the bacteria were then fixed by overnight incubation with 80% isopropanol. Fixed bacteria were coated into multi-well plates at room temperature during overnight, and the ELISA was done by blocking the coated plates with 1% BSA in PBS for 1 h at room temperature. The secondary antibody used was goat anti-mouse IgG, conjugated to HRP (cat #1081-05; Southern Biotech). Detection was achieved by incubation with TMB ELISA substrate (cat# 34029; Thermo) during 10 min at room temperature. In between incubations, plates were washed 5× in an Agilent Biotek TS450 automated plate washer. Absorbances were measured at 450 nm in a BMG Clariostar Plus multimode plate reader.

## RESULTS

### Monoclonal antibody production

A monoclonal antibody (mAb) discovery campaign was conducted to identify novel mAbs targeting bacterial rGAPDH from various species while ensuring no cross-reactivity with human rGAPDH. This campaign, performed as a service by Modiquest (now IPA—ImmunoPrecise Antibodies) using hybridoma technology, led to the identification of a lead candidate, mAb01, which specifically binds to rGAPDH from *Streptococcus pneumoniae* (SP) and *Streptococcus agalactiae* (GBS) without detectable reactivity to human rGAPDH.

**Fig 1:**
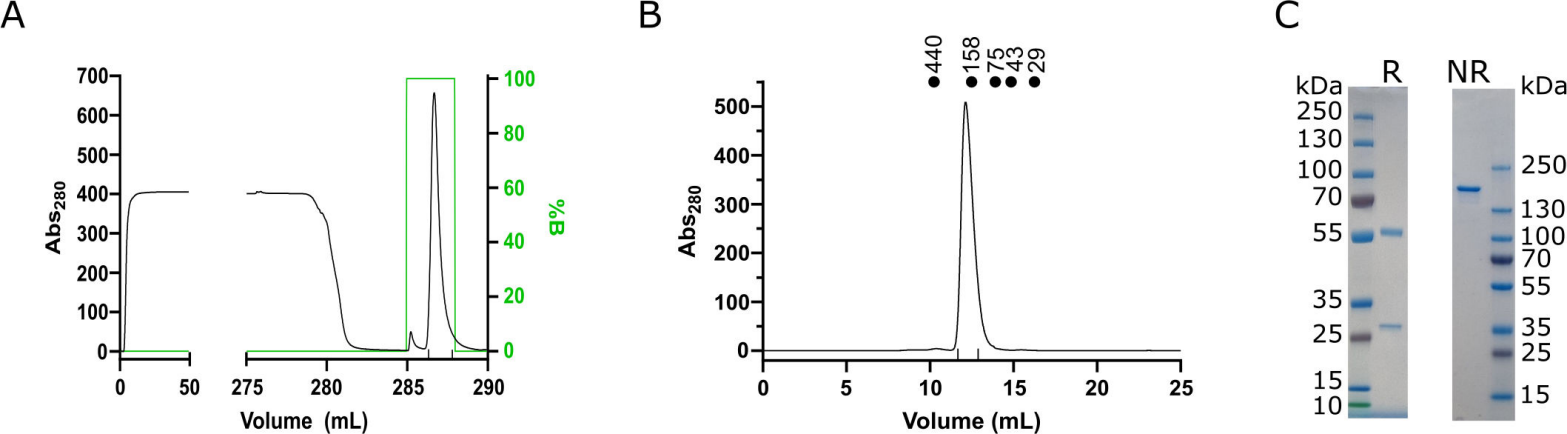
mAb01 purification. (**A**) Protein G affinity chromatography of mAb01 hybridoma cell supernatant. Vertical markers indicate the eluted fractions used. (**B**) Size-exclusion chromatography polishing step of mAb01. Vertical markers indicate the eluted fractions used. The elution of calibration markers is indicated on the top of the chromatogram, ferritin (440 kDa), aldolase (158 kDa), conalbumin (75 kDa), ovalbumin (43 kDa), and carbonic anhydrase (29 kDa). (**C**) SDS-PAGE analysis of purified mAb01 in Tris-Glycine SDS 4%–20% gradient gels in reduced (R) and non-reduced (NR) Laemmli buffer (Bio-Rad). Molecular weights of the standards ladders are indicated.

mAb01 was produced directly from the hybridoma clone in shaking flasks with culture volumes of 50–150 mL. After 5–7 days of incubation in a CO_2_ chamber with agitation, the culture media was centrifuged, filtered, diluted twofold with the same volume of binding buffer (20 mM sodium phosphate + 300 mM NaCL, pH 7.0), and loaded onto a HiTrap Protein G HP 1 mL (Cytiva) using an ÄKTA system. After baseline stabilization, mAb01 was eluted with 0.1 M glycine-HCl, pH 2.7, and collected into appropriate tubes (Fig. 1A). A final polishing step was done by size-exclusion chromatography (SEC) with mAb01 eluting between 12 and 13 mL (Fig. 1B). Based on the column calibration, this retention volume corresponds to a globular protein with an apparent molecular weight of 168 kDa, which is close to the expected molecular weight of mAb01 (147 kDa).

The purified mAb01 was analyzed by SDS-PAGE (Fig. 1C) under non-reducing and reducing conditions and quantified using a Nanodrop spectrophotometer, yielding an average of 30 mg of purified mAb01 per liter of hybridoma culture medium.

**Fig 2:**
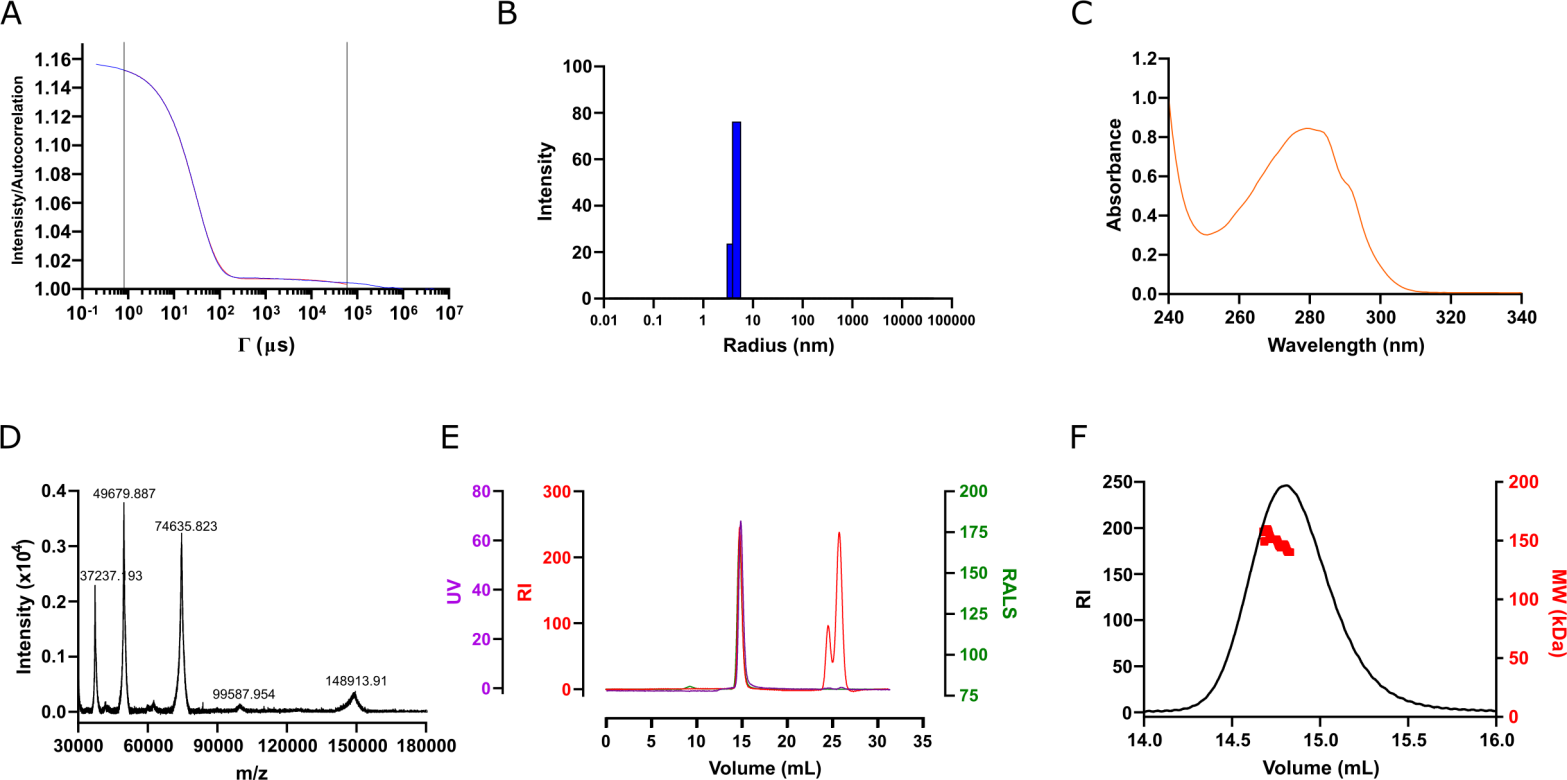
Quality control of purified mAb01. (**A**) DLS raw correlation function. (**B**) DLS corresponding intensity distribution. (**C**) UV-Vis spectrophotometric spectra. (**D**) MALDI-TOF spectra of purified *S. pneumoniae* rGAPDHΔHis. (**E**) SEC-MALS raw data of *S. pneumoniae* rGAPDHΔHis. (**F**) SEC-MALS derived data—detail of the main peak.

The quality of the purified protein was assessed using a set of biophysical techniques (Fig. 2), following the guidelines recommended by the P4EU and ARBRE-MOBIEU European networks (15). The results confirmed the purity and monodispersity of the protein. Dynamic light scattering (DLS) analysis showed that 100% of the protein mass had a hydrodynamic radius of 4.4 nm, with a percentage of polydispersity of 9.8% (Fig. 2A and B), indicating a homogeneous population. UV-Vis spectrophotometry (Fig. 2C) revealed an absorption maximum at 280 nm, as expected for a purified protein, and an *A*_260_/*A*_280_ ratio of 0.51, confirming the absence of nucleic acid contaminants. Additionally, no baseline increase at wavelengths above 310 nm was observed, further indicating the absence of protein aggregation.

The molecular weight of mAb01 was accurately determined by matrix-assisted laser desorption/ionization time-of-flight mass spectrometry (MALDI-TOF MS) (Fig. 2D). The measured molecular weight was 148.9 kDa, with three major peaks at *m*/*z* of 74,636, 49,680, and 37,237, corresponding to di-, tri-, and tetra-charged species of mAb01, respectively. The absence of additional peaks confirmed the lack of contaminants, as previously indicated by UV-Vis and DLS analysis.

The oligomeric state of mAb01 was further evaluated using size-exclusion chromatography coupled with multi-angle light scattering (SEC-MALS) (Fig. 2E and F). The chromatogram confirmed the monodispersity of the purified protein, with a molecular weight of approximately 150 kDa, in agreement with mass spectrometry data. The results demonstrate that mAb01 exists in a monomeric state in solution and meets the expected quality standards.

**Fig 3:**
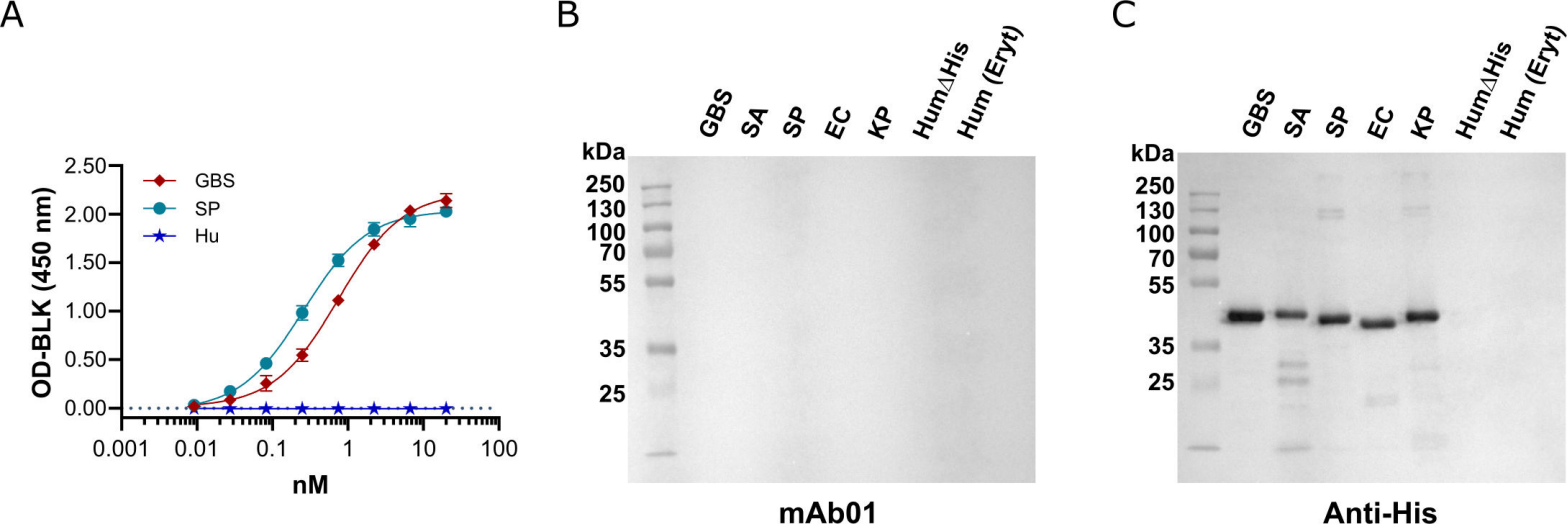
mAb01 interaction with GAPDH. (**A**) Indirect ELISA of mAb01 interaction with recombinant bacterial and human GAPDH. 96-well plates were coated with the corresponding rGAPDH, followed by blocking with BSA. Binding of mAb01 was assessed by a threefold dilution series of mAb01 in duplicate, starting at 3 μg/mL. Detection was accomplished with goat anti-mouse IgG conjugated to horseradish peroxidase. GAPDH detection by western-blot using as primary antibody mAb01 (**B**) or an anti-His tag mAb (**C**). Samples loaded in both gels (**B and C**) were bacterial rGAPDH with C-terminal hexahistidine tags [GBS, SA, SP, EC and KP, tagless human rGAPDH (HumΔHis) and native human GAPDH purified from erythrocytes (Hum(Eryt))].

Following QC validation of both mAb01 (Fig. 2) and the purified rGAPDH proteins (described in the supplemental material), the interaction of mAb01 with bacterial and human rGAPDH proteins was assessed by indirect ELISA (Fig. 3A). The results confirmed the specificity observed during the discovery phase, with an EC_50_ of 0.26 nM for SP rGAPDH produced with a hexahistidine C-terminal tag (rGAPDH_6His) and 0.74 nM for GBS rGAPDH_6His. No binding was detected for human rGAPDH_6His or for rGAPDH_6His from *Staphylococcus aureus* (SA), *Escherichia coli* (EC), or *Klebsiella pneumoniae* (KP) (data not shown), consistent with the initial screening data obtained during the mAb discovery campaign at Modiquest.

To determine whether the mAb01 epitope is linear or conformational, we performed western-blot analysis using mAb01 as the primary antibody, with rGAPDH proteins from human and bacterial species and native GAPDH from human erythrocytes, run under denaturing conditions (Fig. 3B). The absence of detectable bands confirmed that mAb01 recognizes a conformational epitope, which is disrupted upon protein denaturation of the proteins prior to SDS-PAGE. As a control, a parallel western-blot was performed using an anti-histidine tag mAb (THE His tag antibody, Genscript) as the primary antibody (Fig. 3C), which successfully detected all bacterial rGAPDH proteins, confirming their presence and integrity after electrophoresis.

### Kinetic characterization of mAb01 interaction with rGAPDH

**Fig 4:**
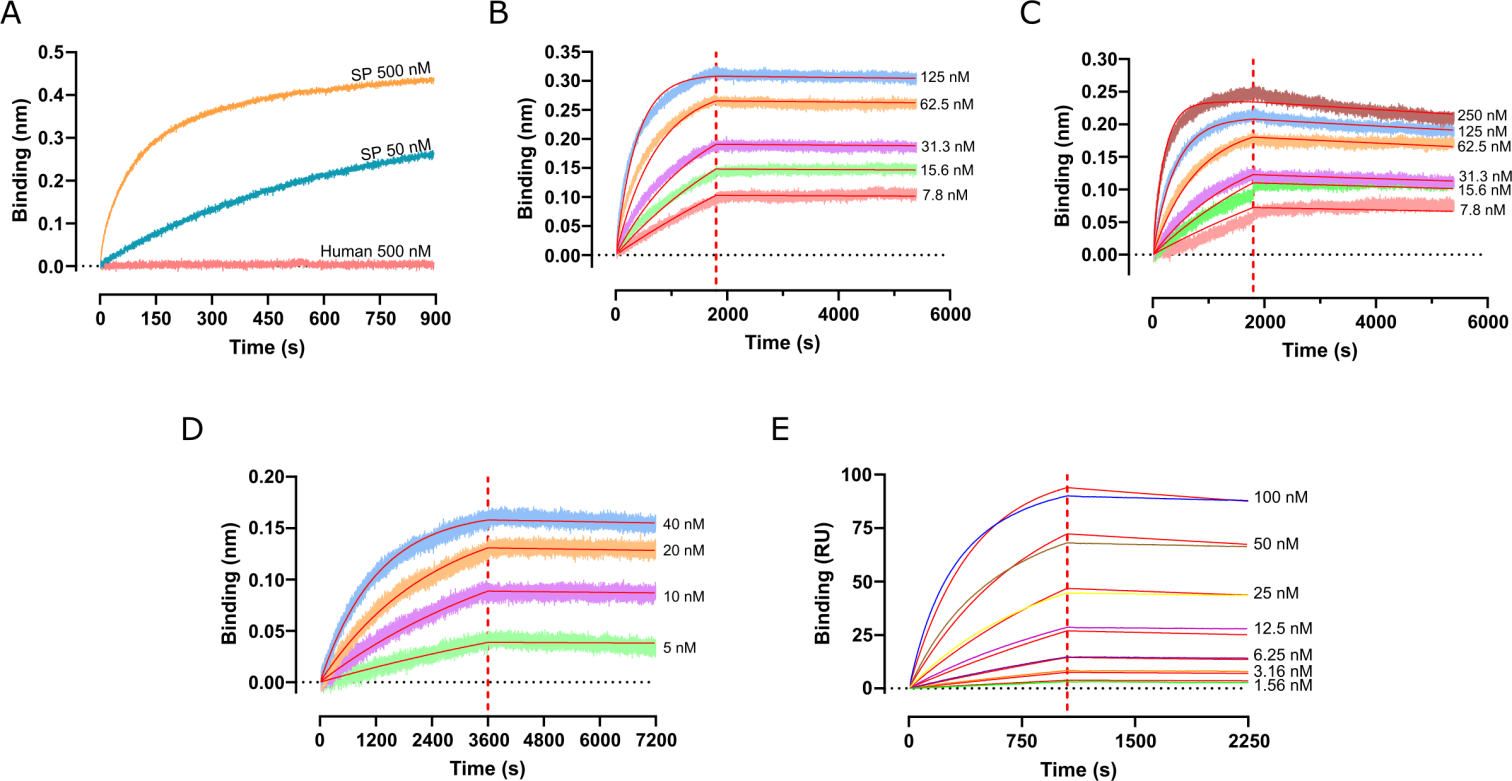
Kinetic characterization of mAb01 interaction with rGAPDH. (**A**) BLI analysis of SP rGAPDHΔHis (50 and 500 nM) and human rGAPDHΔHis (500 nM) binding to captured mAb01 using AMC sensors. (**B, C**) Kinetic characterization of SP and GBS rGAPDHΔHis interactions with captured mAb01 via BLI with AMC sensors. (**D**) BLI-based kinetic analysis of mAb01 binding to captured GBS rGAPDH_6His using Ni²^+^-loaded NTA sensors. (**E**) SPR-based kinetic characterization of mAb01 interaction with captured GBS rGAPDH_6His using an NTA chip.

The kinetics of mAb01 interaction with bacterial rGAPDH proteins were characterized using BLI. Initially, mAb01 was immobilized onto AMC biosensors, and preliminary optimization experiments were conducted to determine the appropriate capture concentrations, association and dissociation parameters, and antigen concentration ranges. As a control, we tested the interaction of mAb01 with human rGAPDHΔHis at 500 nM, confirming that even at this concentration, no binding was observed (Fig. 4A).

We then assessed the binding kinetics of SP rGAPDHΔHis (Fig. 4B) and GBS rGAPDHΔHis (Fig. 4C) to immobilized mAb01. The results indicate a strong preference for SP rGAPDH compared to GBS rGAPDH, as reflected by its faster association rate and slower dissociation rate (Table 1). The dissociation of SP rGAPDHΔHis from captured mAb01 was extremely slow, making an accurate off-rate determination challenging. However, we estimate that the off-rate (*k*_off_) is below 10^−5^ s^−1^, suggesting high binding stability. The calculated affinity values (*K*_D_) were in the single digit nanomolar or even sub-nanomolar range, consistent with the EC_50_ obtained from ELISA.

**TABLE 1:** Kinetic parameters determined by BLI for the interaction of mAb01 with Streptococcus rGAPDH

Ligand	Analyte	Sensor	*K*_D_ (M)	*k*_on_ (M^−1^s^−1^)	*k*_off_ (s^−1^)
mAb01	GBS rGAPDHΔHis	AMC	1.18 × 10^−9^	1.97 × 10^4^	2.34 × 10^−5^
mAb01	SP rGAPDHΔHis	AMC	<4.65 × 10^-10^	2.15 × 10^4^	<1 × 10^−5^
mAb01	GBS rGAPDH_6His	AMC	2.25 × 10^−9^	1.47 × 10^4^	3.31 × 10^−5^
GBS rGAPDH_6His	mAb01	NTA	<5.03 × 10^−10^	1.99 × 10^4^	<1 × 10^−5^

To further investigate the binding characteristics, we reversed the binding orientation and captured GBS rGAPDH_6His on Ni^2+^ loaded NTA biosensors via its C-terminal hexahistidine tag. The kinetic parameters (Fig. 4D and Table 1) were in the same order of magnitude as previously, with only a slightly slower dissociation rate, resulting in increased affinity (*K*_D_ in the order of 0.5 nM). This is consistent with ELISA results, where rGAPDH was also the immobilized binding partner.

To rule out any effect of the hexahistidine tag, we repeated the assay using captured mAb01 and observed similar binding kinetics for GBS rGAPDH_6His and tagless GBS_rGAPDH (Table 1), confirming that the hexahistidine tag does not influence mAb01 binding.

To further validate the binding kinetics, we performed SPR, a technique known for its higher sensitivity and reliability compared to BLI, particularly for high-affinity interactions with low dissociation rates (16). GBS rGAPDH_6His was captured on Ni^2+^ loaded Biacore NTA sensor chips at a density of 80 RU, and a twofold dilution series of mAb01 (100 nM to 1.56 nM) was assayed (Fig. 4E).

The sensorgram data were fitted to a 1:1 Langmuir binding model, yielding a *k*_on_ of 2.71 × 10^4^ M^−1^s^−1^, and a *k*_off_ of 5.05 × 10^−5^ s^−1^, resulting in a dissociation constant (*K*_D_) of 1.86 nM, with a maximum response (*R*_max_) of 96 RU. These results are consistent with the parameters obtained by ELISA and BLI, reinforcing the high affinity of mAb01 for GBS rGAPDH.

### Effect of mAb01 on GAPDH enzymatic activity

To assess whether mAb01 interferes with GAPDH’s glycolytic function, we measured the enzymatic activity of recombinant GAPDH from SP and GBS in the presence or absence of mAb01. GAPDH activity was quantified by monitoring NAD^+^ reduction in a standard coupled assay.

**Fig 5:**
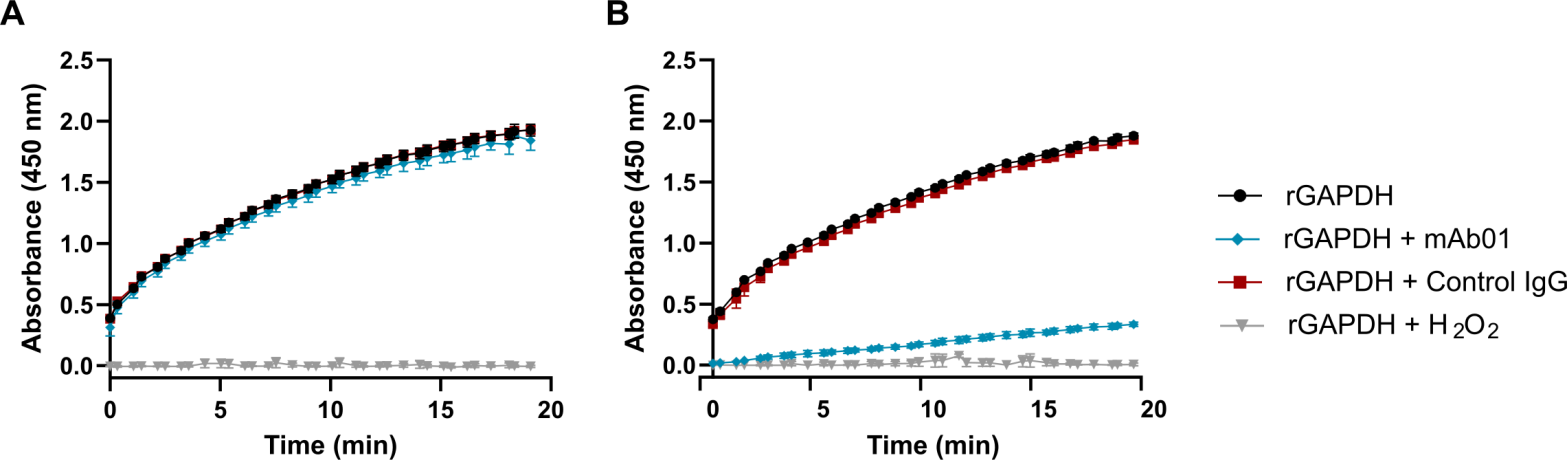
Inhibition of GAPDH enzymatic activity by mAb01. Recombinant GAPDH from GBS (**A**) and *S. pneumoniae* (**B**) was incubated with mAb01 with 16× molar ratio relative to GAPDH tetramer, or with 1 mM H_2_O_2_ to inactivate GAPDH activity. GAPDH enzymatic activity was measured by following NAD^+^ reduction using the GAPDH Activity Assay Kit (Colorimetric) (cat #ab204732) according to manufacturer’s instructions. Data represent mean ± SD from two experimental replicates.

mAb01 significantly inhibited the enzymatic activity of SP GAPDH, whereas no inhibition was observed for GBS GAPDH (Fig. 5). These results indicate species-dependent modulation of catalytic activity. However, previous studies have shown that GAPDH-mediated immunomodulation is independent of enzymatic activity (13) suggesting that the protective effect of mAb01 is not explained by catalytic inhibition.

### Structural modeling of mAb01 interaction with GAPDH

**Fig 6:**
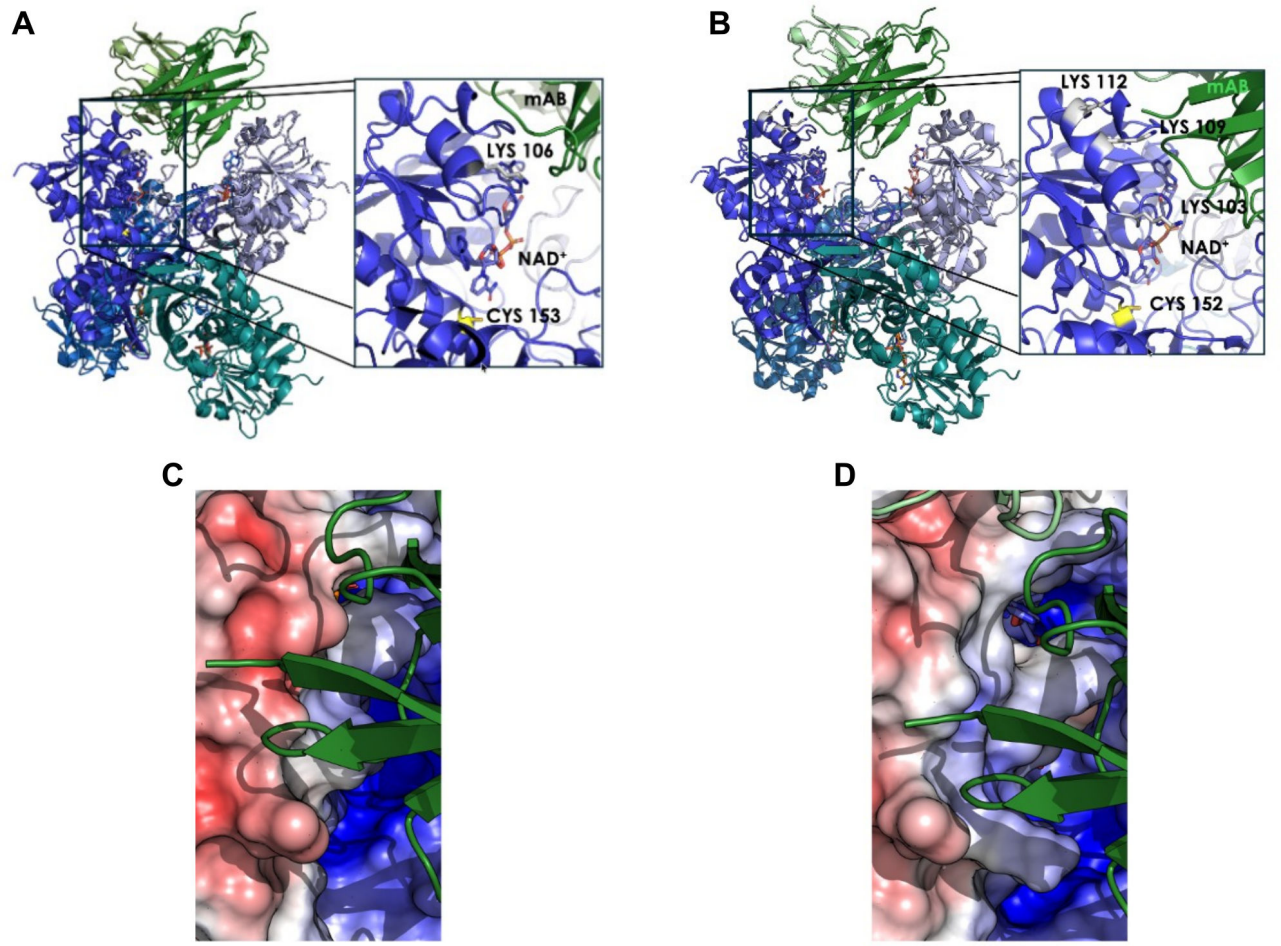
Structural representation of the predicted complexes between the monoclonal antibody (green) and the GAPDH proteins from GBS (**A**) and SP (**B**). Each panel shows a magnified view of the antibody–protein interface, highlighting the NAD^+^ cofactor, the catalytic cysteine, and the lysine residues located within the antibody-contact region of each GAPDH variant (displayed as gray sticks). Panels **C** and **D** show a close-up of the interaction interface for GBS and SP, respectively, overlaid with their electrostatic potential surfaces. In this comparison, the mAb is represented in green cartoon for spatial reference. A markedly more positively charged electrostatic potential is observed in the SP protein at the antibody-binding site, whereas the equivalent region in the GBS protein exhibits a substantially lower positive charge density. This electrostatic contrast provides a structural hypothesis for the differential interaction behavior of the antibody with the two GAPDH variants, which should be experimentally validated.

To gain preliminary insights into the potential binding interface of mAb01, we performed *in silico* docking using OpenFold 3 between the antibody structural model and GAPDH from SP and GBS (Fig. 6).

The predicted complexes should be analyzed with caution, as they lack experimental validation, but suggest that mAb01 binds near the NAD^+^-binding region of GAPDH in both GAPDH variants, a region known to be crucial for catalytic activity (17). Interestingly, although both proteins share approximately 91% sequence identity, the experimental observation that their catalytic activities differ in the presence of the mAb may be rationalized by their distinct electrostatic environments at the antibody-binding interface.

Specifically, the SP protein shows a higher density of positively charged residues in this region, three lysine residues (K103, K109, K112), whereas the GBS protein contains only one lysine (K106) in the equivalent location. These differences likely alter the local electrostatic potential, a key determinant of antibody–antigen affinity and binding mode (18). Therefore, despite their high structural similarity, the electrostatic divergence near the NAD^+^ adjacent mAb-binding region may explain why the SP protein exhibits mAb-mediated inhibition, whereas the GBS protein does not. This interpretation is consistent with reports highlighting how localized charge variations can significantly influence antibody binding and downstream functional effects.

### mAb01 protects neonatal mice from lethal infections caused by different Streptococci

**Fig 7:**
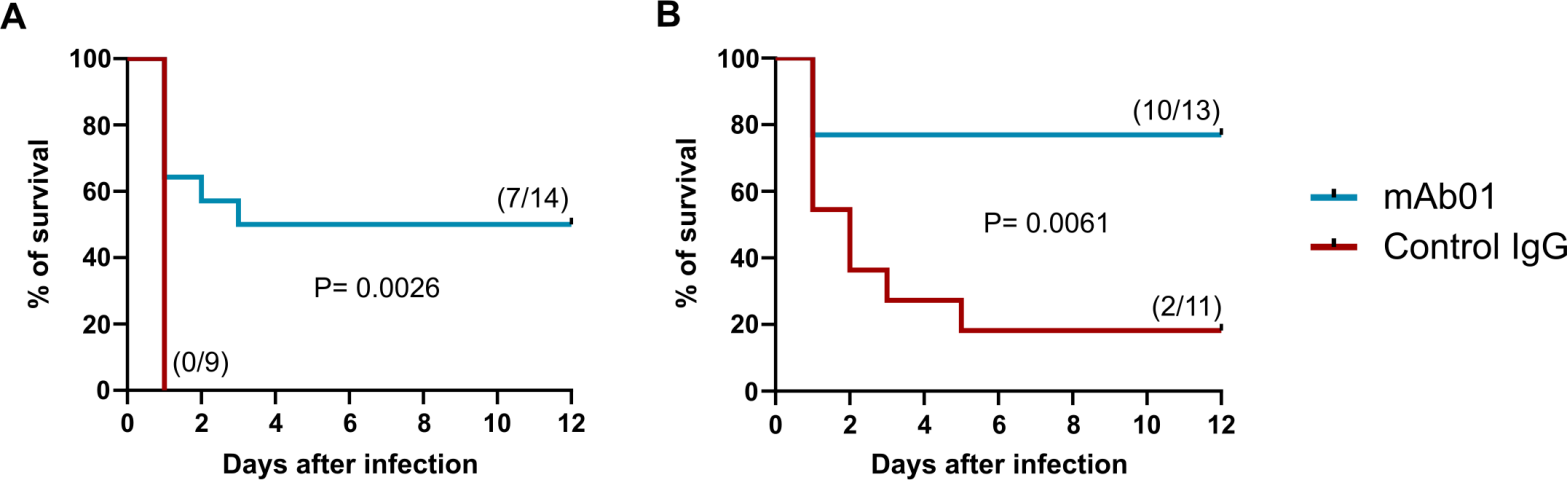
mAb01 protects neonates from GBS and *S. pneumoniae* infections*.* Neonatal C57Bl/6 mice (24 h old) were treated subcutaneously with 200 µg of mAb01 or with an isotype matched control mAb and, 24 h after the passive immunization, were challenged (s.c.) with 3 × 10^6^ CFU of GBS (**A**) and 10^6^ CFU of *S. pneumoniae* (**B**). Treated and control pups were randomly assigned within each cage and left with their mothers during the entire length of the experiment. Numbers in parentheses represent the number of survivors/number of infected pups and graphics represent data from at least five independent experiments. Log-rank test was used to determine differences between groups. *P* values are indicated in the graphs.

We next evaluated whether mAb01 confers protection in a mouse model of sepsis induced by a lethal bacterial inoculum. Neonatal mice were passively immunized with 200 μg of mAb01 or a control mAb, followed by sub-cutaneous infection with a lethal dose of either GBS or SP the next day. GAPDH neutralization by mAb01 resulted in a significant increase in survival of infected mice pups, both for GBS (Fig. 7A) and SP (Fig. 7B), when compared to control-treated animals. Notably, mAb01 exhibited slightly greater efficacy against SP infections than GBS, aligning with its higher binding affinity for SP rGAPDH compared to GBS rGAPDH.

However, caution is required when correlating binding strength to *in vivo* protection, as additional studies are necessary to determine whether mAbs with different kinetic profiles, particularly those targeting the same epitope, exhibit differential protective efficacy.

These findings highlight the potential of mAb01 as a protective therapeutic antibody against neonatal streptococcal infections.

### mAb01 decreases the bacterial burden in an *ex vivo* bacteremia model using human peripheral blood

To further evaluate the efficacy of mAb01 in controlling bacterial infections, we employed an *ex vivo* bacteremia model using human adult peripheral blood (Fig. 6). Peripheral blood samples were incubated with GBS or SP in the presence of 10 μg of mAb01, control IgG, or no antibody as a baseline control. The bacterial inoculum was set at 10-fold the leukocytes count in each blood sample. To assess bacterial load, the colony-forming units (CFU) in the antibody-free condition were set as 100%, and the relative CFU in other conditions were calculated accordingly.

**Fig 8:**
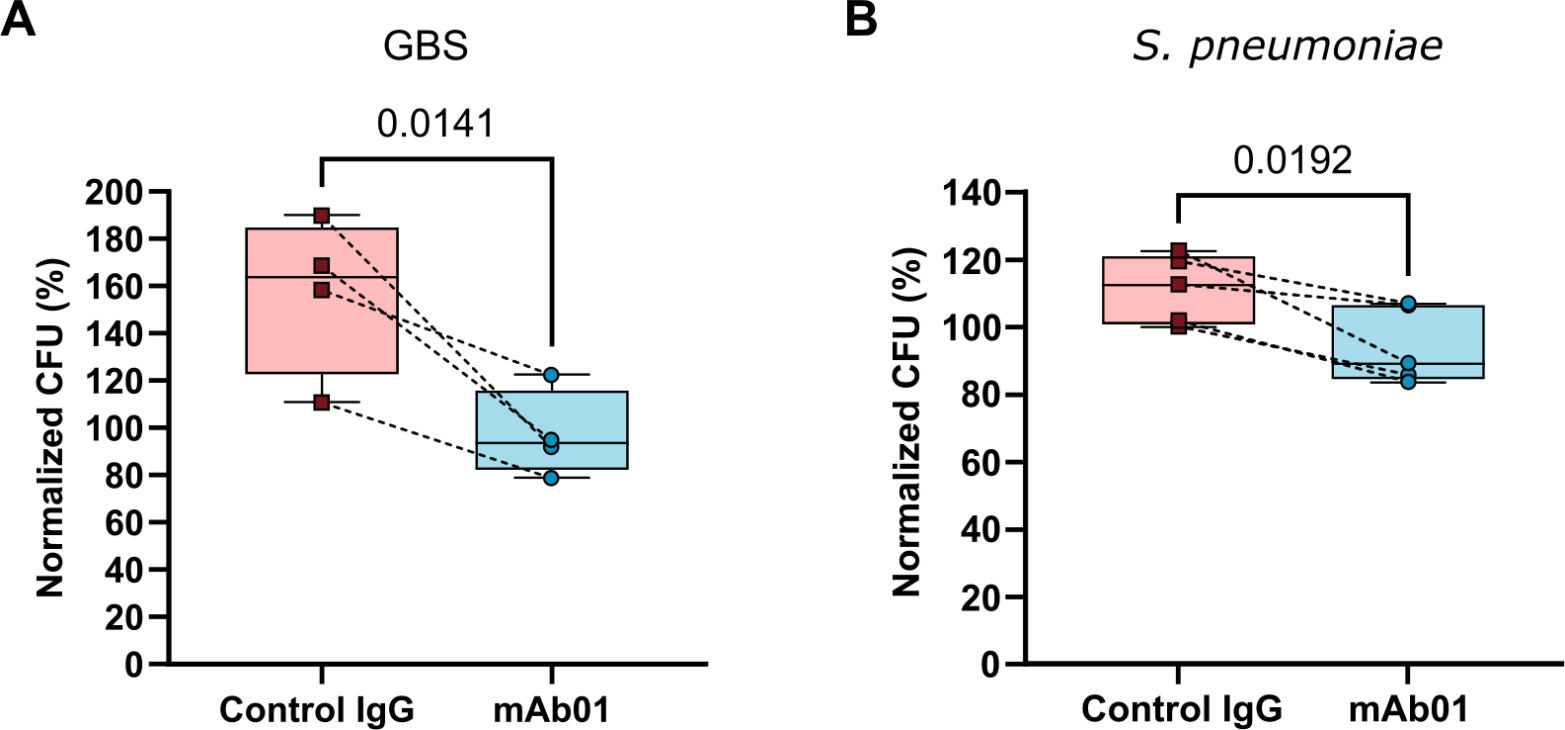
mAb01 controls bacterial replication in adult human peripheral blood*.* Total fresh human peripheral blood from adults was diluted twofold with an equal volume of RPMI medium supplemented with 1% (v/v) 1M HEPES pH 7.2. Diluted blood was incubated with 10 μg of mAb01 (white bars), control IgG (gray bars), or without IgG and infected with GBS (**A**) for 3 h or with *S. pneumoniae* (**B**) for 1 h with a multiplicity of infection of 10, relative to the number of leukocytes. The percentages of bacterial CFU in each indicated condition are relative to the bacterial CFU where no IgG was added to the blood (100%). Results are presented as box plot of at least four independent experiments. Each data point represents an independent biological replicate from a different donor. The dashed line in the box-plot representation corresponds to the same blood sample treated with mAb01 or control IgG. The differences between groups were analyzed using a *t*-test (Mann-Whitney) and a *P* ≤ 0.05 was considered significant.

Treatment with mAb01 resulted in a consistent reduction in bacterial load for GBS and SP when compared to control IgG (Fig. 8), demonstrating its ability to control proliferation in peripheral blood. However, mAb01 appeared to be more effective against GBS than SP, a finding that contrasts with its higher affinity for SP rGAPDH and the superior *in vivo* protection observed for SP infections.

To investigate this discrepancy, we assessed the intrinsic capacity of the human peripheral blood samples to control bacterial growth. Blood samples were incubated with GBS or SP, and the bacterial loads were measured after 1 and 3 h (see Fig. S3). The results revealed that human blood naturally suppresses SP growth more effectively than GBS, with a marked reduction in SP CFU over time. In contrast, GBS bacterial loads showed only a modest decrease. This inherent ability of human blood to control SP bacteremia explains why significant differences in bacterial load were observed only at the 1-h time point in the presence of mAb01, as by 3 h, the natural immune response had already reduced SP levels to a point where additional antibody-mediated effects were no longer detectable.

### GAPDH is detectable on the bacterial surface and recognized by mAb01

GAPDH has been previously reported to localize on the surface of *Streptococcus* species (19–22), where it contributes to adhesion and immune modulation, including IL-10 induction early during infection. While our primary hypothesis is that mAb01 confers protection by neutralizing GAPDH’s immunomodulatory activity, we cannot rule out an alternative or complementary mode of action based on Fc-dependent mechanisms such as opsonophagocytosis or complement-mediated killing.

To confirm that mAb01 can recognize GAPDH in its native context, we performed an ELISA using intact cells of SP and GBS. For this assay, plates were coated with bacteria that had been incubated with porcine IgG to block potential Fc-binding proteins, followed by incubation with mAb01 or an isotype control IgG. After antibody binding, bacteria were fixed using an 80% 2-propanol fixation method, and the detection was performed using an HRP-conjugated secondary antibody.

**Fig 9:**
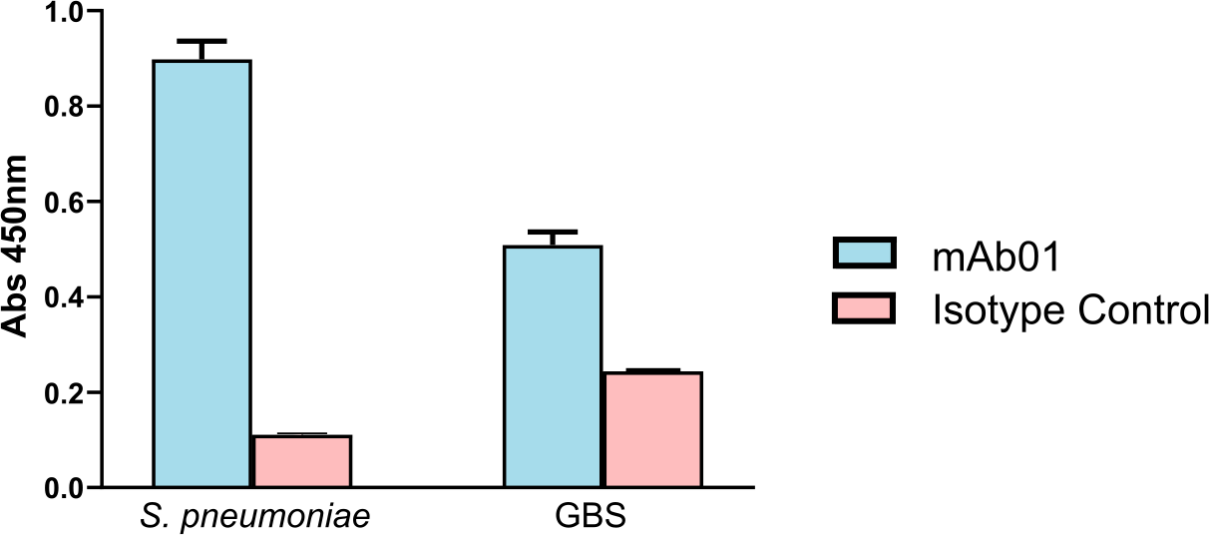
ELISA showing recognition of GAPDH on the surface of *Streptococcus pneumoniae* and GBS by mAb01 compared to isotype control IgG. About 10^7^ CFU of either *S. pneumoniae* or GBS were incubated with swine IgG to block surface Fc-binding receptors and then incubated either with mAb01 or isotype control IgG. After fixation, anti-mouse IgG, HRP-conjugated was used to quantify the amount of antibody at the bacterial surface. Bars represent mean ± SD from two experimental replicates.

The results show that mAb01 binds to the surface of both species, whereas control IgG produced significantly lower signals (Fig. 9). These findings confirm that GAPDH is accessible to mAb01 on the bacterial surface. However, this observation alone does not clarify the mechanism of protection, which may involve Fc-mediated clearance or simply neutralization of bGAPDH to restore the host’s ability to control infection. Future studies will address these possibilities in detail.

## DISCUSSION

In this study, we report the discovery and characterization of a monoclonal antibody, mAb01, targeting GAPDH from GBS and SP. Discovered through hybridoma technology, mAb01 exhibits affinities in the single-digit nanomolar range and provides significant protection in a murine model of lethal bacterial infection. These findings highlight its potential as both a therapeutic and prophylactic agent against streptococcal infections, including those caused by drug-resistant strains. To our knowledge, this is the first reported anti-bGAPDH monoclonal antibody with nanomolar avidity that provides *in vivo* protection against GBS and SP, further advancing previous studies that identified GAPDH as a virulence factor.

The use of mAbs as an alternative to antibiotics offers several advantages, particularly for prophylactic treatment in high-risk patients. To date, the only mAbs approved for bacterial infections target toxins from *Clostridium difficile* (Bezlotoxumab, 2016) or *Bacillus anthracis* (Raxibacumab, 2012; Obilotoxaximab, 2016) (23, 24). Several others are in clinical development, primarily directed against bacterial toxins, virulence factors, conserved surface carbohydrates, and outer membrane proteins from pathogens such as *S. aureus*, *Pseudomonas aeruginosa*, and *Clostridium botulinum* (23, 24). However, no monoclonal antibody specifically targeting *Streptococcus* spp. has reached the clinical stage.

Monoclonal antibodies offer additional advantages, including their highly specific mode of action, which minimizes off-target effects and reduces the risk of resistance. Unlike antibiotics, which broadly affect bacterial populations and disrupt the host microbiome, mAbs can be tailored to selectively neutralize a particular pathogen or antigen. Furthermore, IgG1 mAbs exhibit prolonged serum half-lives of approximately 1 month (25), enabling long-lasting protection with fewer administrations, which is particularly advantageous for prophylaxis in vulnerable populations.

Our findings indicate that mAb01 can inhibit the enzymatic activity of SP GAPDH, while no inhibition was observed for GBS GAPDH. However, this effect is unlikely to explain the protective activity of mAb01, as GAPDH-mediated immunomodulation and IL-10 induction are independent of its glycolytic function (13). This suggests that the primary mechanism of action involves interference with GAPDH’s moonlighting roles rather than catalytic inhibition.

Structural modeling provided additional context: the predicted interface between mAb01 and GAPDH includes a negatively charged pocket in the antibody paratope positioned near positively charged residues on GAPDH. Notably, SP GAPDH contains three lysines in this region, whereas GBS GAPDH has only one. These electrostatic differences may contribute to the slower dissociation rate and the higher affinity observed for SP GAPDH.

It is important to emphasize that these interpretations are based on *in silico* docking and require experimental validation. Future work will include epitope mapping, mutagenesis studies, and mechanistic analyses to confirm the contribution of these structural features to binding and protective efficacy.

The increased survival of mice receiving mAb01 is consistent with previous reports demonstrating protection following vaccination with full-length bGAPDH from GBS and *S. aureus*, as well as with IMTP_vac_1804 (11, 13). The precise mechanism underlying mAb01-mediated protection remains to be elucidated. Future studies are needed to determine whether mAb01 neutralizes bGAPDH by blocking its interaction with TLR2, thereby preventing IL-10-mediated immunosuppression, or whether protection occurs through opsonophagocytic killing. In addition to Fc-mediated clearance, the potential involvement of complement pathways should also be explored, as pneumococcal GAPDH has been reported to interact with C1q without activating the classical complement cascade (26). Understanding whether mAb01 influences these interactions could provide further insight into its mechanism of action.

Although GAPDH has often been described as “secreted,” current evidence indicates that it is primarily released from lysed bacterial cells and subsequently associates with the cell surface, rather than being actively exported through a classical secretion system (21, 26). Given that bGAPDH is known to be released by *Streptococcus* and subsequently deposited on the bacterial cell surface (20, 21, 26, 27), which was also confirmed in this work by the ELISA results, it is plausible that both mechanisms may contribute synergistically to protection. While no direct evidence has yet been gathered to support one mechanism over the other, future studies assessing immune activation markers and phagocytic uptake in the presence of mAb01 will help clarify its mode of action.

A key avenue for future research is determining whether specific epitopes on bGAPDH must be targeted to achieve optimal protection. To address this, a new anti-bGAPDH mAb discovery campaign is underway to generate a broader panel of antibodies targeting multiple epitopes with varying affinities. This will allow us to assess the contribution of epitope specificity and binding strength to protective efficacy. Additionally, the combination of multiple mAbs recognizing different epitopes may enhance protection while further minimizing the potential for bacterial resistance. Although bGAPDH’s essential metabolic role likely constrains its mutational flexibility, alternative bacterial strategies for immune evasion, such as modifying surface interactions, cannot be ruled out. Combining mAb01 with other targeted therapies could further mitigate the potential for resistance development.

The protective effect of mAb01 is expected to extend to additional strains of both SP and GBS. Unlike the surface carbohydrate antigens targeted by existing vaccines and mAbs in clinical development, mAb01 binds to a highly conserved, essential metabolic protein with limited capacity for mutation. This reduces the likelihood of immune escape and resistance. Sequence conservation analyses (see Fig. S4 and S5) further suggest that mAb01 may confer broad protection across all serotypes of these bacteria. Moreover, given that bGAPDH sequences are highly conserved among streptococcal species associated with human disease (82%–99% sequence identity; see Fig. S6), mAb01 may also be effective against other pathogenic *Streptococcus* species.

While this study raises several important questions, the discovery of mAb01 represents a significant advance in the field. Even if potential challenges arise that limit its clinical translation, mAb01 remains a valuable tool for elucidating the role of GAPDH in bacterial virulence and host-pathogen interactions. Future studies will focus on the humanization of mAb01, evaluating intravenous vs subcutaneous administration, and developing optimized formulations. PK/PD modeling will also be incorporated to define dosing strategies and exposure relative to GAPDH levels. In addition, comprehensive safety and functional characterization, including effector function analysis (ADCC, complement activation), neonatal Fc receptor binding, and off-target screening via cell microarrays and tissue cross-reactivity, will be performed to meet regulatory requirements for clinical development. These findings lay the foundation for further preclinical studies and the potential progression of mAb01 to clinical trials.

## SUPPLEMENTAL MATERIAL

Supplemental material aac.00666-25-s0001.docx Fig. S1 and S2; Table S1.

Fig. S3 aac.00666-25-s0002.docx Natural capacity of human blood to control bacteremia.

Supplemental figures aac.00666-25-s0003.docx Fig. S4 to S6.

ASM does not own the copyrights to Supplemental Material that may be linked to, or accessed through, an article. The authors have granted ASM a non-exclusive, world-wide license to publish the Supplemental Material files. Please contact the corresponding author directly for reuse.
