# Supplementary material for: Characterization of a novel monoclonal antibody candidate that targets bacterial GAPDH and protects neonatal mice from infections caused by Streptococcus pneumoniae or Streptococcus agalactiae
Source: Antimicrob Agents Chemother. 2026 Jan 14;70(2):e00666-25. doi: 10.1128/aac.00666-25 (PMC12888860; doi:10.1128/aac.00666-25)
Supplement: Supplemental material — Fig. S1 and S2; Table S1. [file aac.00666-25-s0001.docx]

**Characterization of a novel monoclonal antibody candidate that targets bacterial GAPDH and protects neonatal mice from infections caused by *Streptococcus pneumoniae* or *Streptococcus agalactiae***

**Supplementary Results**

***Recombinant GAPDH Production***

Recombinant glyceraldehyde-3-phosphate dehydrogenase (rGAPDH) proteins, either with N-terminal hexahistidine tag followed by a HRV 3C protease cleavage site or with a C-terminal hexahistidine (6His) tag and no tag at N-terminal, were produced in *Escherichia coli* BL21(DE3)star. The proteins were first purified by IMAC (Immobilized Metal Affinity Chromatography), and the N-terminus 6His tags were cleaved using HRV 3C protease. In both cases, the next purification step involved a preparative size exclusion chromatography (SEC) step. In Fig. S1 it is presented a typical example of the purification workflow, for the purification of *Streptococcus pneumoniae* (SP) rGAPDHΔHis.


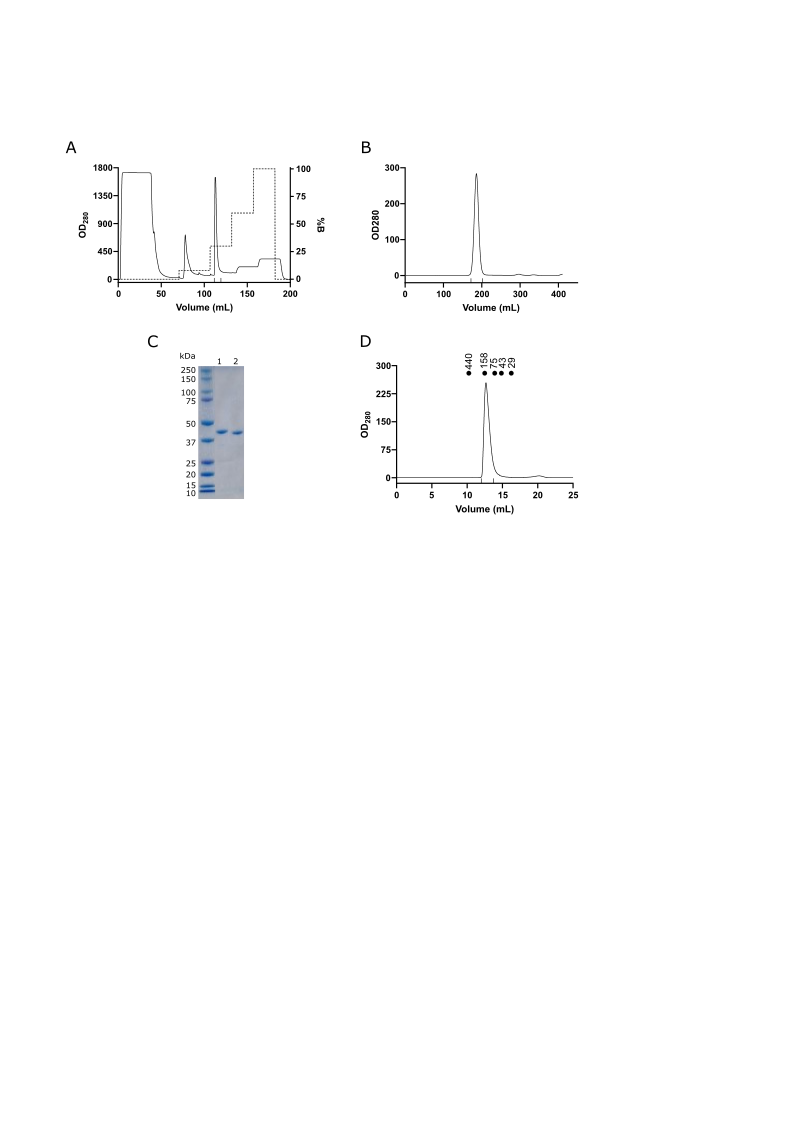


**Fig. S1 – Recombinant SP GAPDHΔHis purification**. **A** – IMAC purification of 6His-3C-rGAPDH using a HisTrap 5 mL (Cytiva) **B** – Size exclusion chromatography of 3C digested SP rGAPDH (SP rGAPDHΔHis) using a Superdex 200 26/600 pg (Cytiva). Protein eluted between 172 and 202 mL (vertical markers). **C** – SDS-PAGE analysis of purified SP rGAPDHΔHis before HRV 3C digestion (Lane 1) and after HRV 3C cleavage and SEC purification (Lane 2). **D** – Polishing size-exclusion chromatography using a Superdex 200 10/300 increase (Cytiva), with collected protein indicated by vertical markers. The elution of calibration markers is indicated on the top of the chromatogram, ferritin (440 kDa), aldolase (158 kDa), conalbumin (75 kDa), ovalbumin (43 kDa) and carbonic anhydrase (29 kDa).

The yields of purified rGAPDH (human rGAPDHΔHis, SP rGAPDHΔHis, GBS rGAPDHΔHis and GBS rGAPDH_6His) varied from 12 - 45 mg of purified protein per litre of expression media at the end of the purification process. The proteins were aliquoted and stored at -80 °C after flash-freeze in liquid nitrogen. When needed, the rGAPDH proteins were thawed at room temperature and a final polishing step was performed by SEC, to remove any aggregates originated from the freeze-thaw process. The purified proteins, after the polishing SEC, were kept at 4 °C and used within 2 weeks.

In the meantime, and prior to the interaction kinetics characterization, protein quality control analysis was performed (Fig. S2). The quality control was done following the P4EU and ARBRE-MOBIEU European networks recommended guidelines, addressing sample homogeneity and quantification by dynamic light scattering (DLS) (Fig. S2 A-B), Protein concentration, DNA contamination and scattering by UV-Vis (at 280, 260 and 340 nm, respectively) (Fig. S2C), integrity of the samples, by MALDI-TOF mass spectrometry, and the oligomerization state of the proteins by SEC-MALS (multiple angle light scattering) (Fig. S2E).

***
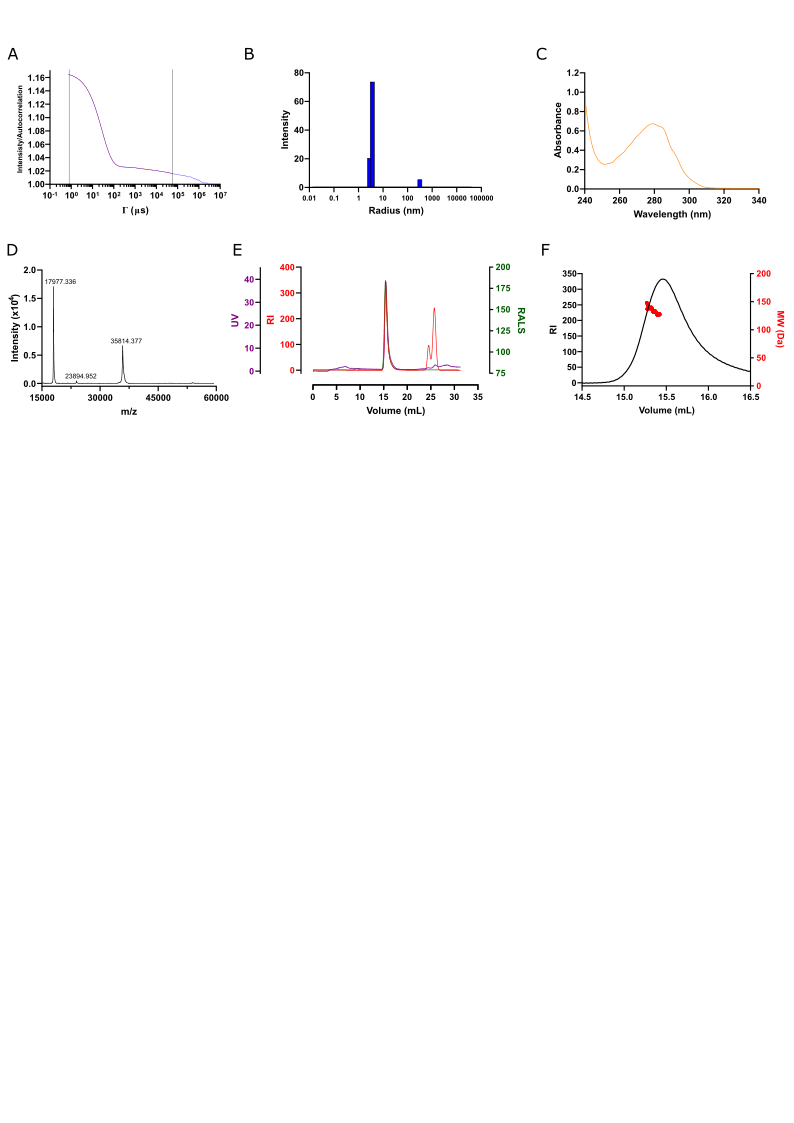
***

**Fig. S2 – Quality control of purified SP rGAPDHΔHis. A –** Dynamic light scattering raw correlation function. **B** – Dynamic light scattering corresponding intensity distribution. **C** – UV-Vis spectrophotometric spectra. **D** – MALDI-TOF spectra of purified SP rGAPDHΔHis. **E –** SEC-MALS raw-data of SP rGAPDHΔHis. **F** – SEC-MALS derived data – detail of the main peak.

The quality control analysis of all the proteins tested, confirmed the high-quality of all the rGAPDH proteins, as can be depicted from Supplementary Table I, below.

Supplementary Table I – Quality control analysis of purified rGAPDH

| Protein | Hydrodynamic radius (DLS) | %Polydispersity | Mass % (DLS) | Molecular Mass (MS) | UV-Vis Concentration | Abs_260/280_ | Molecular weight (MALS) |
| --- | --- | --- | --- | --- | --- | --- | --- |
| SP rGAPDHΔHis | 3.5 nm | 5.1 % | 100 % | 35 884 Da | 1.03 mg/mL | 0.66 | 124 598 Da |
| GBS rGAPDHΔHis | 4.1 nm | 12.3 % | 100 % | 36 034 Da | 1.69 mg/mL | 0.70 | 142 412 Da |
| GBS GAPDH.His | 4.1 nm | 12.4 % | 99.9 % | 37 013 Da | 1.84 mg/mL | 0.69 | 146 627 Da |
| Human GAPDHΔHis | 3.7 nm | 7.7 % | 98.9 % | 36 093 Da | 1.17 mg/mL | 0.79 | 140 994 Da |
