# Supplementary material for: Characterization of a novel monoclonal antibody candidate that targets bacterial GAPDH and protects neonatal mice from infections caused by Streptococcus pneumoniae or Streptococcus agalactiae
Source: Antimicrob Agents Chemother. 2026 Jan 14;70(2):e00666-25. doi: 10.1128/aac.00666-25 (PMC12888860; doi:10.1128/aac.00666-25)
Supplement: Fig. S3 — Natural capacity of human blood to control bacteremia. [file aac.00666-25-s0002.docx]

**Characterization of a novel monoclonal antibody candidate that targets bacterial GAPDH and protects neonatal mice from infections caused by *Streptococcus pneumoniae* or *Streptococcus agalactiae***

**Supplementary Results**


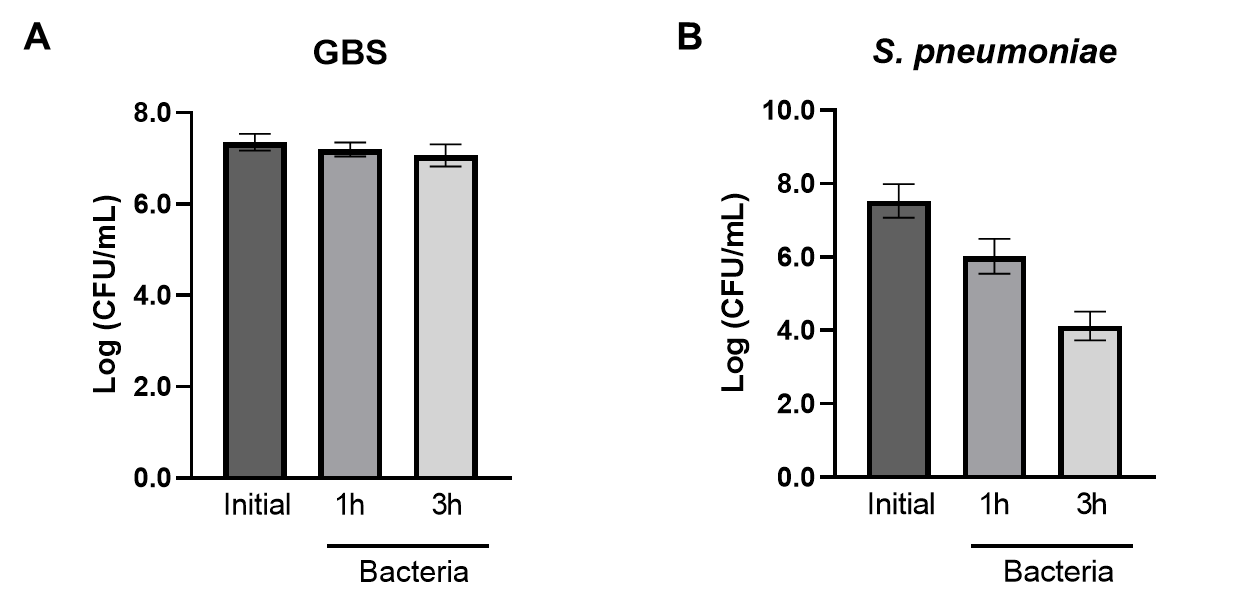


Fig. S3 – *Adult human peripheral blood has a high capacity to control Streptococcus pneumoniae bacteraemia*. Total fresh human peripheral blood from adults was diluted 1:2 in RPMI medium supplemented with 1% (v/v) 1M HEPES pH 7.2. Diluted blood was infected with GBS (A) or with *S. pneumoniae* (B) for 1 h and 3 h with a multiplicity of infection of 10, relative to the number of leucocytes. Bacterial load was determined by measuring the bacterial CFU at the indicated intervals. Each column represents at least 3 experiments.
