# Supplementary material for: Characterization of a novel monoclonal antibody candidate that targets bacterial GAPDH and protects neonatal mice from infections caused by Streptococcus pneumoniae or Streptococcus agalactiae
Source: Antimicrob Agents Chemother. 2026 Jan 14;70(2):e00666-25. doi: 10.1128/aac.00666-25 (PMC12888860; doi:10.1128/aac.00666-25)
Supplement: Supplemental figures — Fig. S4 to S6. [file aac.00666-25-s0003.docx]

**Characterization of a novel monoclonal antibody candidate that targets bacterial GAPDH and protects neonatal mice from infections caused by *Streptococcus pneumoniae* or *Streptococcus agalactiae***

A0AA87CD90_STREE ------------------------MVVKVGINGFGRIGRLAFRRIQNVEGVEVTRINDLT 36

A0A098YV55_STREE ------------------------MVVKVGINGFGRIGRLAFRRIQNVEGVEVTRINDLT 36

Q9AJT6_STREE --------------------------------GFGRIGRLAFRRIQNVEGVEVTRINDLT 28

Q9AJT9_STREE --------------------------------GFGRIGRLAFRRIQNVEGVEVTRINDLT 28

C1CGU4_STRZJ ------------------------MVVKVGINGFGRIGRLAFRRIQNVEGVEVTRINDLT 36

B1I8W5_STRPI ------------------------MVVKVGINGFGRIGRLAFRRIQNVEGVEVTRINDLT 36

A0A654UAP5_STREE ------------------------------------------------------------ 0

I6L8L9_STREE ------------------------MVVKVGINGFGRIGRLAFRRIQNVEGVEVTRINDLT 36

Q9AJT7_STREE --------------------------------GFGRIGRLAFRRIQNVEGVEVTRINDLT 28

Q9AJT8_STREE ---------------------------------FGRIGRLAFRRIQNVEGVEVTRINDLT 27

A0A0H2US80_STRPN ------------------------MVVKVGINGFGRIGRLAFRRIQNVEGVEVTRINDLT 36

A0A0H2ZNS0_STRP2 ------------------------MVVKVGINGFGRIGRLAFRRIQNVEGVEVTRINDLT 36

Q8CWN6_STRR6 MICSDSSYSFHNKNFMIFIRRKSLMVVKVGINGFGRIGRLAFRRIQNVEGVEVTRINDLT 60

Q9AJT4_STREE --------------------------------GFGRIGRLAFRRIQNVEGVEVTRINDLT 28

A0A9P1QE27_STREE ------------------------MVVKVGINGFGRIGRLAFRRIQSVEGVEVTRINDLT 36

A0A0E8JNM6_STREE ------------------------MVVKVGINGFGRIGRLAFRRIQNVEGVEVTRINDLT 36

Q9AJT5_STREE --------------------------------GFGRIGRLAFRRIQNVEGVEVTRINDLT 28

C1CA58_STRP7 ------------------------MVVKVGINGFGRIGRLAFRRIQNVEGVEVTRINDLT 36

B2IMI7_STRPS MICSDSSYSFHNKNFMIFIRRKSLMVVKVGINGFGRIGRLAFRRIQNVEGVEVTRINDLT 60

A0AA87CD90_STREE DPVMLAHLLKYDTTQGRFDGTVEVKEGGFEVNGKFVKVSAERDPEQIDWATDGVEIVLEA 96

A0A098YV55_STREE DPVMLAHLLKYDTTQGRFDGTVEVKEGGFEVNGKFVKVSAERDPEQIDWATDGVEIVLEA 96

Q9AJT6_STREE DPVMLAHLLKYDTTQGRFDGTVEVKEGGFEVNGKFVKVSAERDPEQIDWATDGVEIVLEA 88

Q9AJT9_STREE DPVMLAHLLKYDTTQGRFDGTVEVKEGGFEVNGKFVKVSAERDPEQIDWATDGVEIVLEA 88

C1CGU4_STRZJ DPVMLAHLLKYDTTQGRFDGTVEVKEGGFEVNGKFVKVSAERDPEQIDWATDGVEIVLEA 96

B1I8W5_STRPI DPVMLAHLLKYDTTQGRFDGTVEVKEGGFEVNGKFVKVSAERDPEQIDWATDGVEIVLEA 96

A0A654UAP5_STREE ---MLAHLLKYDTTQGRFDGTVEVKEGGFEVNGKFIKVSAERDPEQIDWATDGVEIVLEA 57

I6L8L9_STREE DPVMLAHLLKYDTTQGRFDGTVEVKEGGFEVNGKFIKVSAERDPEQIDWATDGVEIVLEA 96

Q9AJT7_STREE DPVMLAHLLKYDTTQGRFDGTVEVKEGGFEVNGKFIKVSAERDPEQIDWATDGVEIVLEA 88

Q9AJT8_STREE DPVMLAHLLKYDTTQGRFDGTVEVKEGGFEVNGKFIKVSAERDPEQIDWATDGVEIVLEA 87

A0A0H2US80_STRPN DPVMLAHLLKYDTTQGRFDGTVEVKEGGFEVNGKFIKVSAERDPEQIDWATDGVEIVLEA 96

A0A0H2ZNS0_STRP2 DPVMLAHLLKYDTTQGRFDGTVEVKEGGFEVNGKFIKVSAERDPEQIDWATDGVEIVLEA 96

Q8CWN6_STRR6 DPVMLAHLLKYDTTQGRFDGTVEVKEGGFEVNGKFIKVSAERDPEQIDWATDGVEIVLEA 120

Q9AJT4_STREE DPVMLAHLLKYDTTQGRFDGTVEVKEGGFEVNGKFIKVSAERDPEQIDWATDGVEIVLEA 88

A0A9P1QE27_STREE DPVMLAHLLKYDTTQGRFDGTVEVKEGGFEVNGKFIKVSAERDPEQIDWATDGVEIVLEA 96

A0A0E8JNM6_STREE DPVMLAHLLKYDTTQGRFDGTVEVKEGGFEVNGKFIKVSAERDPEQIDWATDGVEIVLEA 96

Q9AJT5_STREE DPVMLAHLLKYDTTQGRFDGTVEVKEGGFEVNGKFIKVSAERDPEQIDWATDGVEIVLEA 88

C1CA58_STRP7 DPVMLAHLLKYDTTQGRFDGTVEVKEGGFEVNGKFIKVSAERDPEQIDWATDGVEIVLEA 96

B2IMI7_STRPS DPVMLAHLLKYDTTQGRFDGTVEVKEGGFEVNGKFIKVSAERDPEQIDWATDGVEIVLEA 120

********************************:************************

A0AA87CD90_STREE TGFFAKKEAAEKHLKGGAKKVVITAPGGNDVKTVVFNTNHDVLDGTETVISGASCTTNCL 156

A0A098YV55_STREE TGFFAKKEAAEKHLKGGAKKVVITAPGGNDVKTVVFNTNHDVLDGTETVISGASCTTNCL 156

Q9AJT6_STREE TGFFAKKEAAEKHLKGGAKKVVITAPGGNDVKTVVFNTNHDVLDGTETVISGASCTTNCL 148

Q9AJT9_STREE TGFFAKKEAAEKHLKGGAKKVVITAPGGNDVKTVVFNTNHDVLDGTETVISGASCTTNCL 148

C1CGU4_STRZJ TGFFAKKEAAEKHLKGGAKKVVITAPGGNDVKTVVFNTNHDVLDGTETVISGASCTTNCL 156

B1I8W5_STRPI TGFFAKKEAAEKHLKGGAKKVVITAPGGNDVKTVVFNTNHDVLDGTETVISGASCTTNCL 156

A0A654UAP5_STREE TGFFAKKEAAEKHLKGGAKKVVITAPGGNDVKTVVFNTNHDVLDGTETVISGASCTTNCL 117

I6L8L9_STREE TGFFAKKEAAEKHLKGGAKKVVITAPGGNDVKTVVFNTNHDVLDGTETVISGASCTTNCL 156

Q9AJT7_STREE TGFFAKKEAAEKHLKGGAKKVVITAPGGNDVKTVVFNTNHDVLDGTETVISGASCTTNCL 148

Q9AJT8_STREE TGFFAKKEAAEKHLKGGAKKVVITAPGGNDVKTVVFNTNHDVLDGTETVISGASCTTNCL 147

A0A0H2US80_STRPN TGFFAKKEAAEKHLKGGAKKVVITAPGGNDVKTVVFNTNHDVLDGTETVISGASCTTNCL 156

A0A0H2ZNS0_STRP2 TGFFAKKEAAEKHLKGGAKKVVITAPGGNDVKTVVFNTNHDVLDGTETVISGASCTTNCL 156

Q8CWN6_STRR6 TGFFAKKEAAEKHLKGGAKKVVITAPGGNDVKTVVFNTNHDVLDGTETVISGASCTTNCL 180

Q9AJT4_STREE TGFFAKKEAAEKHLKGGAKKVVITAPGGNDVKTVVFNTNHNVLDGTETVISGASCTTNCL 148

A0A9P1QE27_STREE TGFFAKKEAAEKHLKGGAKKVVITAPGGNDVKTVVFNTNHNVLDGTETVISGASCTTNCL 156

A0A0E8JNM6_STREE TGFFAKKEAAEKHLKGGAKKVVITAPGGNDVKTVVFNTNHNVLDGTETVISGASCTTNCL 156

Q9AJT5_STREE TGFFAKKEAAEKHLKGGAKKVVITAPGGNDVKTVVFNTNHNVLDGTETVISGASCTTNCL 148

C1CA58_STRP7 TGFFAKKEAAEKHLKGGAKKVVITAPGGNDVKTVVFNTNHNVLDGTETVISGASCTTNCL 156

B2IMI7_STRPS TGFFAKKEAAEKHLKGGAKKVVITAPGGNDVKTVVFNTNHNVLDGTETVISGASCTTNCL 180

****************************************:*******************

A0AA87CD90_STREE APMAKALQDNFGVVEGLMTTIHAYTGDQMILDGPHRGGDLRRARAGAANIVPNSTGAAKA 216

A0A098YV55_STREE APMAKALQDNFGVVEGLMTTIHAYTGDQMILDGPHRGGDLRRARAGAANIVPNSTGAAKA 216

Q9AJT6_STREE APMAKALQDNFGVVEGLMTTIHAYTGDQMILDGPHRGGDLRRARAGAANIVPNSTGAAKA 208

Q9AJT9_STREE APMAKALQDNFGVVEGLMTTIHAYTGDQMILDGPHRGGDLRRARAGAANIVPNSTGAAKA 208

C1CGU4_STRZJ APMAKALQDNFGVVEGLMTTIHAYTGDQMILDGPHRGGDLRRARAGAANIVPNSTGAAKA 216

B1I8W5_STRPI APMAKALQDNFGVVEGLMTTIHAYTGDQMILDGPHRGGDLRRARAGAANIVPNSTGAAKA 216

A0A654UAP5_STREE APMAKALQDNFGVVEGLMTTIHAYTGDQMILDGPHRGGDLRRARAGAANIVPNSTGAAKA 177

I6L8L9_STREE APMAKALQDNFGVVEGLMTTIHAYTGDQMILDGPHRGGDLRRARAGAANIVPNSTGAAKA 216

Q9AJT7_STREE APMAKALQDNFGVVEGLMTTIHAYTGDQMILDGPHRGGDLRRARAGAANIVPNSTGAAKA 208

Q9AJT8_STREE APMAKALQDNFGVVEGLMTTIHAYTGDQMILDGPHRGGDLRRARAGAANIVPNSTGAAKA 207

A0A0H2US80_STRPN APMAKALQDNFGVVEGLMTTIHAYTGDQMILDGPHRGGDLRRARAGAANIVPNSTGAAKA 216

A0A0H2ZNS0_STRP2 APMAKALQDNFGVVEGLMTTIHAYTGDQMILDGPHRGGDLRRARAGAANIVPNSTGAAKA 216

Q8CWN6_STRR6 APMAKALQDNFGVVEGLMTTIHAYTGDQMILDGPHRGGDLRRARAGAANIVPNSTGAAKA 240

Q9AJT4_STREE APMAKALQDNFGVVEGLMTTIHAYTGDQMILDGPHRGGDLRRARAGAANIVPNSTGAAKA 208

A0A9P1QE27_STREE APMAKALQDNFGVVEGLMTTIHAYTGDQMILDGPHRGGDLRRARAGAANIVPNSTGAAKA 216

A0A0E8JNM6_STREE APMAKALQDNFGVVEGLMTTIHAYTGDQMILDGPHRGGDLRRARAGAANIVPNSTGAAKA 216

Q9AJT5_STREE APMAKALQDNFGVVEGLMTTIHAYTGDQMILDGPHRGGDLRRARAGAANIVPNSTGAAKA 208

C1CA58_STRP7 APMAKALQDNFGVVEGLMTTIHAYTGDQMILDGPHRGGDLRRARAGAANIVPNSTGAAKA 216

B2IMI7_STRPS APMAKALQDNFGVVEGLMTTIHAYTGDQMILDGPHRGGDLRRARAGAANIVPNSTGAAKA 240

************************************************************

A0AA87CD90_STREE IGLVIPELNGKLDGSAQRVPTPTGSVTELVAVLEKNVTVDEVNAAMKAASNESYGYTEDP 276

A0A098YV55_STREE IGLVIPELNGKLDGSAQRVPTPTGSVTELVAVLEKNVTVDEVNAAMKAASNESYGYTEDP 276

Q9AJT6_STREE IGLVIPELNGKLDGSAQRVPTPTGSVTELVAVLEKNVTVDEVNAAMKAASNESYGYTEDP 268

Q9AJT9_STREE IGLVIPELNGKLDGSAQRVPTPTGSVTELVAVLEKNVTVDEVNAAMKAASNESYGYTEDP 268

C1CGU4_STRZJ IGLVIPELNGKLDGSAQRVPTPTGSVTELVAVLEKNVTVDEVNAAMKAASNESYGYTEDP 276

B1I8W5_STRPI IGLVIPELNGKLDGSAQRVPTPTGSVTELVAVLEKNVTVDEVNAAMKAASNESYGYTEDP 276

A0A654UAP5_STREE IGLVIPELNGKLDGSAQRVPTPTGSVTELVAVLEKNVTVDEVNAAMKAASNESYGYTEDP 237

I6L8L9_STREE IGLVIPELNGKLDGSAQRVPTPTGSVTELVAVLEKNVTVDEVNAAMKAASNESYGYTEDP 276

Q9AJT7_STREE IGLVIPELNGKLDGSAQRVPTPTGSVTELVAVLEKNVTVDEVNAAMKAASNESYGYTEDP 268

Q9AJT8_STREE IGLVIPELNGKLDGSAQRVPTPTGSVTELVAVLEKNVTVDEVNAAMKAASNESYGYTEDP 267

A0A0H2US80_STRPN IGLVIPELNGKLDGSAQRVPTPTGSVTELVAVLEKNVTVDEVNAAMKAASNESYGYTEDP 276

A0A0H2ZNS0_STRP2 IGLVIPELNGKLDGSAQRVPTPTGSVTELVAVLEKNVTVDEVNAAMKAASNESYGYTEDP 276

Q8CWN6_STRR6 IGLVIPELNGKLDGSAQRVPTPTGSVTELVAVLEKNVTVDEVNAAMKAASNESYGYTEDP 300

Q9AJT4_STREE IGLVIPELNGKLDGSAQRVPTPTGSVTELVAVLEKNVTVDEVNAAMKAASNESYGYTEDP 268

A0A9P1QE27_STREE IGLVIPELNGKLDGSAQRVPTPTGSVTELVAVLEKNVTVDEVNAAMKAASNESYGYTEDP 276

A0A0E8JNM6_STREE IGLVIPELNGKLDGSAQRVPTPTGSVTELVAVLEKNVTVDEVNAAMKAASNESYGYTEDP 276

Q9AJT5_STREE IGLVIPELNGKLDGSAQRVPTPTGSVTELVAVLEKNVTVDEVNAAMKAASNESYGYTEDP 268

C1CA58_STRP7 IGLVIPELNGKLDGSAQRVPTPTGSVTELVAVLEKNVTVDEVNAAMKAASNESYGYTEDP 276

B2IMI7_STRPS IGLVIPELNGKLDGSAQRVPTPTGSVTELVAVLEKNVTVDEVNAAMKAASNESYGYTEDP 300

************************************************************

A0AA87CD90_STREE IVSSDIIGMSYGSLFDATQTKVLDVDGKQLVKVVSWYDNEMSYTAQLVRTLEYFAKIAK 335

A0A098YV55_STREE IVSSDIVGMSYGSLFDATQTKVLDVDGKQLVKVVSWYDNEMSYTAQLVRTLEYFAKIAK 335

Q9AJT6_STREE IVSSDIVGMSYGSLFDATQTKVLDVDGKQLVKVVSWY---------------------- 305

Q9AJT9_STREE IVSSDIVGMSYGSLFDATQTKVLDVDGKQLVKVVSWYDNEM------------------ 309

C1CGU4_STRZJ IVSSDIVGMSYGSLFDATQTKVLDVDGKQLVKVVSWYDNEMSYTAQLVRTLEYFAKIAK 335

B1I8W5_STRPI IVSSDIVGMSYGSLFDATQTKVLDVDGKQLVKVVSWYDNEMSYTAQLVRTLEYFAKIAK 335

A0A654UAP5_STREE IVSSDIVGMSYGSLFDATQTKVLDVDGKQLVKVVSWYDNEMSYTAQLVRTLEYFAKIAK 296

I6L8L9_STREE IVSSDIVGMSYGSLFDATQTKVLDVDGKQLVKVVSWYDNEMSYTAQLVRTLEYFAKIAK 335

Q9AJT7_STREE IVSSDIVGMSYGSLFDATQTKVLDVDGKQLVKVVSWYDNEM------------------ 309

Q9AJT8_STREE IVSSDIVGMSYGSLFDATQTKVLDVDGKQLVKVVSWYDNEM------------------ 308

A0A0H2US80_STRPN IVSSDIVGMSYGSLFDATQTKVLDVDGKQLVKVVSWYDNEMSYTAQLVRTLEYFAKIAK 335

A0A0H2ZNS0_STRP2 IVSSDIVGMSYGSLFDATQTKVLDVDGKQLVKVVSWYDNEMSYTAQLVRTLEYFAKIAK 335

Q8CWN6_STRR6 IVSSDIVGMSYGSLFDATQTKVLDVDGKQLVKVVSWYDNEMSYTAQLVRTLEYFAKIAK 359

Q9AJT4_STREE IVSSDIVGISYGSLFDATQTKVLDVDGKQLVKVVSWYDNEM------------------ 309

A0A9P1QE27_STREE IVSSDIVGMSYGSLFDATQTKVLDVDGKQLVKVVSWYDNEMSYTAQLVRTLEYFAKIAK 335

A0A0E8JNM6_STREE IVSSDIVGMSYGSLFDATQTKVLDVDGKQLVKVVSWYDNEMSYTAQLVRTLEYFAKIAK 335

Q9AJT5_STREE IVSSDIVGMSYGSLFDATQTKVLDVDGKQLVKVVSWYDNEM------------------ 309

C1CA58_STRP7 IVSSDIVGMSYGSLFDATQTKVLDVDGKQLVKVVSWYDNEMSYTAQLVRTLEYFAKIAK 335

B2IMI7_STRPS IVSSDIVGMSYGSLFDATQTKVLDVDGKQLVKVVSWYDNEMSYTAQLVRTLEYFAKIAK 359

******:*:****************************

**Fig. S4 – Protein sequence alignment of the bGAPDH sequences from *Streptococcus* *pneumoniae* available at Uniprot.** Multiple protein sequence alignment of GAPDH obtained using Clustal Omega (1.2.4) at Uniprot. An “*” (asterisk) indicates positions which have a single, fully conserved residue; a “:” (colon) indicates conservation between groups of residues with strongly similar properties, and a “.” (period) indicates conservation between groups of residues with weakly similar properties.

V6Z2W6_STRAG MTVKVGINGFGRIGRLAFRRIQNVEGVEVARINDLTDPAMLAHLLKYDTTQGRFDGDVEV 60

Q8KHG1_STRAG MVVKVGINGFGRIGRLAFRRIQNVEGVEVTRINDLTDPNMLAHLLKYDTTQGRFDGTVEV 60

Q4H1F8_STRAG MVVKVGINGFGRIGRLAFRRIQNVEGVEVTRINDLTDPNMLAHLLKYDTTQGRFDGTVEV 60

A0A0H1UZQ7_STRAG MVVKVGINGFGRIGRLAFRRIQNVEGVEVTRINDLTDPNMLAHLLKYDTTQGRFDGTVEV 60

A0A829IA39_STRAG MVVKVGINGFGRIGRLAFRRIQNVEGVEVTRINDLTDPNMLAHLLKYDTTQGRFDGTVEV 60

Q9ALW2_STRAG MVVKVGINGFGRIGRLAFRRIQNVEGVEVTRINDLTDPNMLAHLLKYDTTQGRFDGTVEV 60

Q8E3E8_STRA3 MVVKVGINGFGRIGRLAFRRIQNVEGVEVTRINDLTDPNMLAHLLKYDTTQGRFDGTVEV 60

Q8DXS8_STRA5 MVVKVGINGFGRIGRLAFRRIQNVEGVEVTRINDLTDPNMLAHLLKYDTTQGRFDGTVEV 60

*.***************************:******** ***************** ***

V6Z2W6_STRAG KDNGFEVNGKFVKVSAERDPEQIDWAADGVEIVLEATGFFTSKEGAEKHIHENGAKKVVI 120

Q8KHG1_STRAG KDGGFEVNGSFVKVSAEREPANIDWATDGVDIVLEATGFFASKAAAEQHIHANGAKKVVI 120

Q4H1F8_STRAG KEGGFEVNGQFVKVSAEREPANIDWATDGVEIVLEATGFFASKEKAEQHIHENGAKKVVI 120

A0A0H1UZQ7_STRAG KEGGFEVNGQFVKVSAEREPANIDWATDGVEIVLEATGFFASKEKAEQHIHENGAKKVVI 120

A0A829IA39_STRAG KEGGFEVNGQFVKVSAEREPANIDWATDGVEIVLEATGFFASKEKAEQHIHENGAKKVVI 120

Q9ALW2_STRAG KEGGFEVNGQFVKVSAEREPANIDWATDGVEIVLEATGFFASKEKAEQHIHENGAKKVVI 120

Q8E3E8_STRA3 KEGGFEVNGQFVKVSAEREPANIDWATDGVEIVLEATGFFASKEKAEQHIHENGAKKVVI 120

Q8DXS8_STRA5 KEGGFEVNGQFVKVSAEREPANIDWATDGVEIVLEATGFFASKEKAEQHIHENGAKKVVI 120

*:.******.********:* :****:***:*********:** **:*** ********

V6Z2W6_STRAG TAPGGNDVKTVVFNTNHDILDGTETVISAGSCTTNCLAPMADTLNKAFGLKVGTMTTIHG 180

Q8KHG1_STRAG TAPGGNDVKTVVYNTNHDILDGTETVISGASCTTNCLAPMAKALQDNFGVKQGLMTTIHG 180

Q4H1F8_STRAG TAPGGNDVKTVVFNTNHDILDGTETVISGASCTTNCLAPMAKALQDNFGVKQGLMTTIHA 180

A0A0H1UZQ7_STRAG TAPGGNDVKTVVFNTNHDILDGTETVISGASCTTNCLAPMAKALQDNFGVKQGLMTTIHA 180

A0A829IA39_STRAG TAPGGNDVKTVVFNTNHDILDGTETVISGASCTTNCLAPMAKALQDNFGVKQGLMTTIHA 180

Q9ALW2_STRAG TAPGGNDVKTVVFNTNHDILDGTETVISGASCTTNCLAPMAKALQDNFGVKQGLMTTIHA 180

Q8E3E8_STRA3 TAPGGNDVKTVVFNTNHDILDGTETVISGASCTTNCLAPMAKALQDNFGVKQGLMTTIHA 180

Q8DXS8_STRA5 TAPGGNDVKTVVFNTNHDILDGTETVISGASCTTNCLAPMAKALQDNFGVKQGLMTTIHA 180

************:***************..***********.:*:. **:* * *****.

V6Z2W6_STRAG YTGDQMTLDGPHRGGDFRRARAAAENIIPNSTGAAKAIGLVIPELNGKLKGHAQRVPVPT 240

Q8KHG1_STRAG YTGDQMVLDGPHRGGDLRRARAAAANIVPNSTGAAKAIGLVIPELNGKLDGAAQRVPVPT 240

Q4H1F8_STRAG YTGDQMILDGPHRGGDLRRARAGAANIVPNSTGAAKAIGLVIPELNGKLDGAAQRVPVPT 240

A0A0H1UZQ7_STRAG YTGDQMILDGPHRGGDLRRARAGAANIVPNSTGAAKAIGLVIPELNGKLDGAAQRVPVPT 240

A0A829IA39_STRAG YTGDQMILDGPHRGGDLRRARAGAANIVPNSTGAAKAIGLVIPELNGKLDGAAQRVPVPT 240

Q9ALW2_STRAG YTGDQMILDGPHRGGDLRRARAGAANIVPNSTGAAKAIGLVIPELNGKLDGAAQRVPVPT 240

Q8E3E8_STRA3 YTGDQMILDGPHRGGDLRRARAGAANIVPNSTGAAKAIGLVIPELNGKLDGAAQRVPVPT 240

Q8DXS8_STRA5 YTGDQMILDGPHRGGDLRRARAGAANIVPNSTGAAKAIGLVIPELNGKLDGAAQRVPVPT 240

****** *********:*****.* **:*********************.* ********

V6Z2W6_STRAG GSLTELVSVLDKKVTEEEVNEAMKAATTESYGFTTDQIVSSDIVGMSFGSLFDATQTEIT 300

Q8KHG1_STRAG GSVTELVAVLEKDTSVEEINAAMKAAANDSYGYTEDAIVSSDIVGISYGSLFDATQTKVQ 300

Q4H1F8_STRAG GSVTELVETLEKDVTVEEVNAAMKAAANDSYGYTEDPIVSSAYRGISYGSLFDATQTKVQ 300

A0A0H1UZQ7_STRAG GSVTELVATLGKDVTVEEVNAAMKAAANDSYGYTEDPIVSSDIVGISYGSLFDATQTKVQ 300

A0A829IA39_STRAG GSVTELVATLGKDVTVEEVNAAMKAAANDSYGYTEDPIVSSDIVGISYGSLFDATQTKVQ 300

Q9ALW2_STRAG GSVTELVATLEKDVTVEEVNAAMKAAANDSYGYTEDPIVSSDIVGISYGSLFDATQTKVQ 300

Q8E3E8_STRA3 GSVTELVATLEKDVTVEEVNAAMKAAANDSYGYTEDPIVSSDIVGISYGSLFDATQTKVQ 300

Q8DXS8_STRA5 GSVTELVATLEKDVTVEEVNAAMKAAANDSYGYTEDPIVSSDIVGISYGSLFDATQTKVQ 300

**:**** .* *..: **:* *****:.:***:* * **** *:*:*********::

V6Z2W6_STRAG EAEDGTQLVKTVSWYDNEMSYTSQLVRTLEYFAKIAK 337

Q8KHG1_STRAG -TVDGNQLVKVVSWYDNEMSYTAQLVRTLEYFAKIAK 336

Q4H1F8_STRAG -TVDGNQLVKVVSWYDNEMSYTSKLVRTLEYFAKIAK 336

A0A0H1UZQ7_STRAG -TVDGNQLVKVVSWYDNEMSYTSQLVRTLEYFAKIAK 336

A0A829IA39_STRAG -TVDGNQLVKVVSWYDNEMSYTSQLVRTLEYFAKIAK 336

Q9ALW2_STRAG -TVDGNQLVKVVSWYDNEMSYTSQLVRTLEYFAKIAK 336

Q8E3E8_STRA3 -TVDGNQLVKVVSWYDNEMSYTSQLVRTLEYFAKIAK 336

Q8DXS8_STRA5 -TVDGNQLVKVVSWYDNEMSYTSQLVRTLEYFAKIAK 336

: **.****.***********::*************

**Fig. S5 – Protein sequence alignment of the bGAPDH from *Streptococcus* *agalactiae* available at Uniprot.** Multiple protein sequence alignment of GAPDH obtained using Clustal Omega (1.2.4) at Uniprot. Sequences A0A829IA39, A0A0H1UZQ7 and V6Z2W6 appear at Uniprot tagged as preliminary data from whole genome shotgun.An “*” (asterisk) indicates positions which have a single, fully conserved residue; a “:” (colon) indicates conservation between groups of residues with strongly similar properties, and a “.” (period) indicates conservation between groups of residues with weakly similar properties.

*S. gallolyticus* (WP_074628069) mvvkvgingfgrigrlafrriqnvegvevarindltdpamlahllkydttqgrfdgdvvv 60

*S. agalactiae* (WP_165696333.1) mvvkvgingfgrigrlafrriqnvegvevtrindltdpnmlahllkydttqgrfdgtvev 60

*S. pyogenes* (WP_136116906.1) mvvkvgingfgrigrlafrriqniegvevtrindltdpnmlahllkydttqgrfdgtvev 60

*S. dysgalactiae* (WP_110408172.1) mvvkvgingfgrigrlafrriqnvegvevtrindltdpnmlahllkydttqgrfdgtvev 60

*S. mutans* (WP_002270329.1) mvvkvgingfgrigrlafrriqnvegvevtrindltdpnmlahllkydstqgrfdgnvev 60

*S. sanguinis* (WP_125351477.1) mvvkvgingfgrigrlafrriqnvegvevtrindltdpvmlahllkydttqgrfdgtvev 60

*S. anginosus* (WP_003036557.1) mvvkvgingfgrigrlafrriqnvegvevtrindltdpvmlahllkydttqgrfdgtvev 60

*S. pneumoniae* (WP_000260665.1) mvvkvgingfgrigrlafrriqnvegvevtrindltdpvmlahllkydttqgrfdgtvev 60

*S. mitis* (WP_142559283.1) mvvkvgingfgrigrlafrriqnvegvevtrindltdpvmlahllkydttqgrfdgtvev 60

***********************:*****:******** *********:******* * *

*S. gallolyticus* (WP_074628069) kdggfevngkfvkvsaerdpeqidwandgveivleatgffatkaaaekhlhe-ggakkvv 119

*S. agalactiae* (WP_165696333.1) keggfevngqfvkvsaerepanidwatdgveivleatgffaskekaeqhiheng-akkvv 119

*S. pyogenes* (WP_136116906.1) keggfevngnfikvsaerdpenidwatdgveivleatgffakkeaaekhlhang-akkvv 119

*S. dysgalactiae* (WP_110408172.1) keggfevngnfikvsaerdpenidwatdgveivleatgffakkeaaekhlhang-akkvv 119

*S. mutans* (WP_002270329.1) keggfevngkfvkvsaerdpeqidwavdgveivleatgffaskaaaekhlhanggakkvv 120

*S. sanguinis* (WP_125351477.1) keggfevngkfvkvsaerdpeqidwatdgveivleatgffakkdaaekhlk--ggakkvv 118

*S. anginosus* (WP_003036557.1) keggfevngkfvkvsaerdpeqidwatdgveivleatgffakkdaaekhlk--ggakkvv 118

*S. pneumoniae* (WP_000260665.1) keggfevngkfikvsaerdpeqidwatdgveivleatgffakkeaaekhlk--ggakkvv 119

*S. mitis* (WP_142559283.1) keggfevngkfvkvsaerdpeqidwatdgveivleatgffakkeaaekhlha-ggakkvv 119

*:*******:*:******:* :**** **************.* **:*:: * *****

*S. gallolyticus* (WP_074628069) itapggsdvktivfntnheildgtetvisagscttnclapmadalnksfglkvgtmttvh 179

*S. agalactiae* (WP_165696333.1) itapggndvktvvfntnhdildgtetvisgascttnclapmakalqdnfgvkqglmttih 179

*S. pyogenes* (WP_136116906.1) itapggndvktvvfntnhdildgtetvisgascttnclapmakalhdtfgiqkglmttih 179

*S. dysgalactiae* (WP_110408172.1) itapggndvktvvfntnhdildgtetvisgascttnclapmakalhdafgiqkglmttih 179

*S. mutans* (WP_002270329.1) itapggndiktivfntnhdvldgtetvisgascttnclapmakalhdnfsikeglmttih 180

*S. sanguinis* (WP_125351477.1) itapggndvktivfntnhdildgtetvisgascttnclapmakalqdnfgvveglmttih 178

*S. anginosus* (WP_003036557.1) itapggndvktivfntnhdvldgtetvisgascttnclapmakalqdnfgiveglmttih 178

*S. pneumoniae* (WP_000260665.1) itapggndvktvvfntnhdvldgtetvisgascttnclapmakalqdnfgvveglmttih 179

*S. mitis* (WP_142559283.1) itapggndvktivfntnhdildgtetvisgascttnclapmakalqdnfgvveglmttih 179

******.*:**:******::*********..***********.**:. *.: * ***:*

*S. gallolyticus* (WP_074628069) gytgdqmtldaphrkgdfrraraaaenivpnstgaakaiglvipelngklqghaqrvpvp 239

*S. agalactiae* (WP_165696333.1) aytgdqmildgphrggdlrraragaanivpnstgaakaiglvipelngkldgaaqrvpvp 239

*S. pyogenes* (WP_136116906.1) aytgdqmildgphrggdlrraragaanivpnstgaakaiglvipelngkldgaaqrvpvp 239

*S. dysgalactiae* (WP_110408172.1) aytgdqmildgphrggdlrraragaanivpnstgaakaiglvipelngkldgaaqrvpvp 239

*S. mutans* (WP_002270329.1) aytgdqmvldgphrkgdlrraraaaanivpnstgaakaiglvipelngkldgaaqrvpvp 240

*S. sanguinis* (WP_125351477.1) aytgdqmildgphrggdlrraragaanivpnstgaakaiglvipelngkldgsaqrvptp 238

*S. anginosus* (WP_003036557.1) aytgdqmildgphrkgdlrraragaanivpnstgaakaiglvipelngkldgsaqrvptp 238

*S. pneumoniae* (WP_000260665.1) aytgdqmildgphrggdlrraragaanivpnstgaakaiglvipelngkldgsaqrvptp 239

*S. mitis* (WP_142559283.1) aytgdqmildgphrggdlrraragaanivpnstgaakaiglvipelngkldgsaqrvptp 239

.****** **.*** **:*****.* ************************:* *****.*

*S. gallolyticus* (WP_074628069) tgsltelvsvldkkvtaeevnaamqaaatesfgyntdpivsrdivgisfgslfdatqtev 299

*S. agalactiae* (WP_165696333.1) tgsvtelvatlekdvtveevnaamkaaandsygytedpivssdivgisygslfdatqtkv 299

*S. pyogenes* (WP_136116906.1) tgsvtelvvtldknvsvdeinaamkaasndsfgytedpivssdivgvsygslfdatqtkv 299

*S. dysgalactiae* (WP_110408172.1) tgsvtelvvtldkdvsvdeinaamkaasndsfgytedpivssdivgvsygslfdatqtkv 299

*S. mutans* (WP_002270329.1) tgsvtelvavldkkvtvdevnaamkaaanesygytedpivssdivgmsfgslfdatqtkv 300

*S. sanguinis* (WP_125351477.1) tgsvtelvavleknitvdevnaamkaaanesygytedpivssdivgmaygslfdatqtkv 298

*S. anginosus* (WP_003036557.1) tgsvtelvavleknvtvdevnaamkaaanesygytedpivssdivgmaygslfdatqtkv 298

*S. pneumoniae* (WP_000260665.1) tgsvtelvavleknvtvdevnaamkaasnesygytedpivssdivglsygslfdatqtkv 299

*S. mitis* (WP_142559283.1) tgsvtelvavleknvtvdevnaamkaaanesygytedqivssdivgmaygslfdatqtkv 299

***:**** .*:*.::.:*:****:**:.:*:**. * *** ****:::*********:*

*S. gallolyticus* (WP_074628069) teaadgtqlvktvswydnemsytsqlvrtleyfakiak 337

*S. agalactiae* (WP_165696333.1) Qt-idgnqlvkvvswydnemsytsqlvrtleyfakiak 336

*S. pyogenes* (WP_136116906.1) me-vdgsqlvkvvswydnemsytaqlvrtleyfakiak 336

*S. dysgalactiae* (WP_110408172.1) me-vdgsqlvkvvswydnemsytaqlvrtleyfakiak 336

*S. mutans* (WP_002270329.1) ld-vdgkqlvkvvswydnemsytsqlvrtleyfakiak 337

*S. sanguinis* (WP_125351477.1) ld-vdgkqlvkvvswydnemsytaqlvrtleyfakiak 335

*S. anginosus* (WP_003036557.1) ld-vdgkqlvkvvswydnemsytaqlvrtleyfakiak 335

*S. pneumoniae* (WP_000260665.1) ld-vdgkqlvkvvswydnemsytaqlvrtleyfakiak 336

*S. mitis* (WP_142559283.1) ld-vdgkqlvkvvswydnemsytaqlvrtleyfakiak 336

**.****.***********:**************

**Fig. S6 – Protein sequence alignment of GAPDH from the most common *Streptococcus* species causing human disease.** Multiple protein sequence alignment of GAPDH obtained using Clustal Omega (1.2.4) at Uniprot. Each protein ID indicates the species and the NCBI ID. An “*” (asterisk) indicates positions which have a single, fully conserved residue; a “:” (colon) indicates conservation between groups of residues with strongly similar properties, and a “.” (period) indicates conservation between groups of residues with weakly similar properties.
